# Dual-loop upcycling of spent LiFePO_4_: defect inheritance enables durable and fast-charging sodium-ion batteries

**DOI:** 10.1093/nsr/nwaf321

**Published:** 2025-08-08

**Authors:** Xiao-Tong Wang, Zhen-Yi Gu, Jun-Ming Cao, Xin-Xin Zhao, Han-Hao Liu, Shuo-Hang Zheng, Yong-Li Heng, Kai-Yang Zhang, Edison Huixiang Ang, Zhe Wang, Ronghua Zeng, Xing-Long Wu

**Affiliations:** State Key Laboratory of Integrated Optoelectronics, MOE Key Laboratory for UV Light-Emitting Materials and Technology, Northeast Normal University, Changchun 130024, China; State Key Laboratory of Integrated Optoelectronics, MOE Key Laboratory for UV Light-Emitting Materials and Technology, Northeast Normal University, Changchun 130024, China; State Key Laboratory of Integrated Optoelectronics, MOE Key Laboratory for UV Light-Emitting Materials and Technology, Northeast Normal University, Changchun 130024, China; Department of Chemistry, Northeast Normal University, Changchun 130024, China; Department of Chemistry, Northeast Normal University, Changchun 130024, China; State Key Laboratory of Integrated Optoelectronics, MOE Key Laboratory for UV Light-Emitting Materials and Technology, Northeast Normal University, Changchun 130024, China; State Key Laboratory of Integrated Optoelectronics, MOE Key Laboratory for UV Light-Emitting Materials and Technology, Northeast Normal University, Changchun 130024, China; State Key Laboratory of Integrated Optoelectronics, MOE Key Laboratory for UV Light-Emitting Materials and Technology, Northeast Normal University, Changchun 130024, China; Natural Sciences and Science Education, National Institute of Education, Nanyang Technological University, Singapore 637616, Singapore; Guangdong Provincial International Joint Research Center for Energy Storage Materials, School of Chemistry, South China Normal University, Guangzhou 510006, China; Guangdong Provincial International Joint Research Center for Energy Storage Materials, School of Chemistry, South China Normal University, Guangzhou 510006, China; State Key Laboratory of Integrated Optoelectronics, MOE Key Laboratory for UV Light-Emitting Materials and Technology, Northeast Normal University, Changchun 130024, China

**Keywords:** spent lithium-ion batteries, LiFePO_4_, sodium-ion batteries, cathode, Na_2_FeP_2_O_7_

## Abstract

The growing accumulation of spent lithium-ion batteries (LIBs) presents pressing environmental and societal challenges, highlighting the urgent need to reimagine them as sustainable energy resources. Traditionally, the formation of Fe vacancies ($V_{Fe}^{^{\prime\prime}}$) in LiFePO_4_ (LFP) cathodes during extended cycling has been regarded as the chief culprit contributing to capacity degradation. However, this study uncovers their functional potential as beneficial structural defects for sodium-ion batteries, repurposing $V_{Fe}^{^{\prime\prime}}$ from spent LFP batteries to engineer high-performance Na–Fe–P–O series cathode materials. These pre-existing vacancies trigger a self-adaptive lattice breathing mechanism that dynamically accommodates volume changes during rapid Na^+^ ion de-/intercalation, achieving 80% state-of-charge within 6 min and retaining 82.9% capacity after 4000 cycles at a high rate of 10 C. The proposed dual-loop upcycling model further enhances economic returns by 65% and reduces environmental footprint by 29%. This work pioneers a sustainable paradigm that transforms degradation mechanisms of LIBs into foundational design strategies for next-generation batteries.

## Teaser

A sustainable dual-loop recycling strategy has been proposed, aiming to utilize structural vacancies derived from spent LiFePO4 cathodes to facilitate fast-charging Na-storage cathodes for sodium-ion batteries.

## INTRODUCTION

Driven by the exponential growth in electric vehicle (EV) adoption, global demand for lithium-ion batteries (LIBs) is surging. Among these, LiFePO_4_ (LFP) batteries have emerged as the dominant chemistry, projected to account for over 40% of global EV battery demand by 2023—more than double their market share in 2020 [1,2]. As these batteries approach the end of their life, large-scale decommissioning poses pressing challenges for sustainable resource recovery. Recycling has thus become critical to mitigate the environmental impact of raw material extraction and reduce the volume of critical elements relegated to landfills [3]. However, despite recent advances in LIB recycling technologies, the field continues to lack a globally scalable, commercially viable and environmentally responsible framework. This gap highlights a persistent misalignment with the United Nations Sustainable Development Goal (SDG) 7, which emphasizes ensuring access to affordable, reliable, sustainable and modern energy for all [4–8]. To accelerate the transition toward clean energy systems, developing closed-loop, circular recycling strategies is imperative for enhancing energy security, reducing environmental footprint and fostering long-term sustainability in the battery industry.

Current academic consensus attributes the degradation of LFP cathodes primarily to the irreversible formation of Li vacancies ($V_{Li}^{\prime}$), Fe vacancies ($V_{Fe}^{^{\prime\prime}}$) and Li–Fe anti-site ($Fe_{Li}^ \cdot $) defects [9–11]. These crystallographic imperfections progressively disrupt electron conduction pathways and hinder Li^+^ diffusion kinetics, ultimately leading to capacity fade [12]. Paradoxically, recent advances in sodium-ion battery (SIB) systems research reveal a contrasting paradigm—vacancy engineering is increasingly recognized as a beneficial strategy to enhance electrochemical performance and alleviate structural collapse in various Na-storage electrode materials during fast charging and discharging [12–15]. Such engineered vacancies facilitate improved charge transport and mitigate volumetric strain. For instance, Hou *et al.* developed a defective cathode material, Na_3.2_□_0.8_Co_0.5_Fe_0.5_V(PO_3.9_F_0.1_)_3_ (where □ denotes a Na vacancy), via fluoride ion doping. The introduced vacancies activate reversible and rapid Na⁺ ion de-/intercalation, enabling high energy and power densities. These vacancies serve as host sites for the redox activity of V^4+^/V^5+^ at elevated voltages while suppressing structural deformation, thereby enhancing both structural integrity and energy density [16]. Similarly, Kim *et al.* demonstrated that inducing Na deficiencies stabilizes the rose polymorph of Na_2_CoP_2_O_7_, which exhibits superior cycling stability and an average voltage of 4.3 V versus Na^+^/Na, surpassing its blue polymorph counterpart in energy density [17]. Zhao *et al.* achieved pure-phase Na_4_Fe_3_(PO_4_)_2_P_2_O_7_ (NFPP) by incorporating a controlled amount of Fe^2+^ ion vacancies ($V_{Fe}^{^{\prime\prime}}$) during synthesis [18]. Hu *et al.* further demonstrated that modulating $V_{Fe}^{^{\prime\prime}}$ effectively suppresses the formation of NaFePO_4_ impurity phases in NFPP, enhancing structural adaptability and mitigating volumetric strain during electrochemical cycling [19]. These local lattice distortions alleviate mechanical stress during Na^+^ ion de-/intercalation, thereby significantly improving cycle stability. Collectively, these studies findings underscore how vacancy-induced lattice distortions reduce migration energy barrier and promote Na^+^ ion diffusion. Importantly, they also highlight a fundamental shift in the materials science perspective: structural defects traditionally viewed as detrimental in LIB systems may possess unique and advantageous electrochemical functions in SIB architectures.

Herein, we propose and demonstrate a defect inheritance strategy that transforms degradation pathways from the LIB era into design principles for robust SIBs. Specifically, the self-adaptive $V_{Fe}^{^{\prime\prime}}$ inherited from spent LFP cathodes serve dual functions: buffering volumetric fluctuations and providing additional Na⁺ storage sites. Using density functional theory calculations in conjunction with X-ray absorption fine structure analysis, we show that $V_{Fe}^{^{\prime\prime}}$ induce Fe 3*d*-orbital energy splitting and generate localized electron-rich states *via* Fe–O coordination distortion. These effects collectively reduce the charge transfer energy barrier to as little as 0.003 eV. Taking Na_2_FeP_2_O_7_ (NFPO) as an example, we demonstrate that this defect-engineered cathode delivers exceptional fast-charging performance, achieving 80% state-of-charge within only 6 min and retaining 82.9% of its initial capacity after 4000 cycles at a high rate of 10 C. This vacancy engineering strategy is also extendable to other Na–Fe–P–O cathode chemistries, underscoring its broad applicability. Importantly, leveraging intrinsic $V_{Fe}^{^{\prime\prime}}$ defects derived from end-of-life LFP cathodes eliminates the need for energy-intensive artificial defect synthesis, enhancing both environmental and economic sustainability. Beyond materials innovation, we further introduce a dual-loop recycling framework, offering a novel ‘failure-to-functionality’ approach that not only upcycles spent LIB materials but also advances the sustainable design of next-generation SIB cathodes in alignment with circular economy principles.

## RESULTS AND DISCUSSION

### Monitoring structural transformation

**Figure 1.**
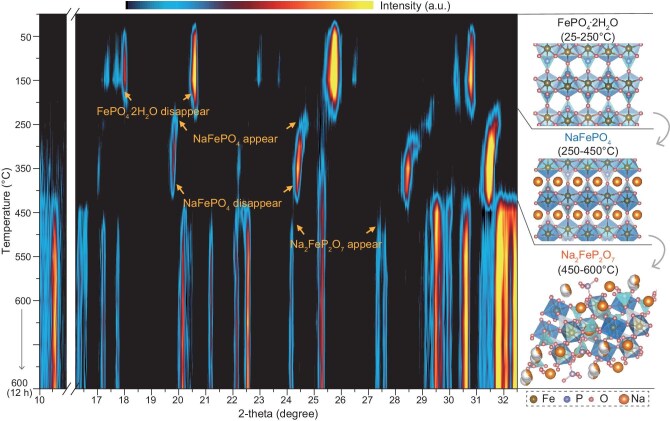
Physical phase evolution monitoring. *In situ* variable temperature XRD contour map and the schematic illustration of crystal structural transformation. The yellow contour indicates high intensity and the black contour indicates low intensity.

As shown in Fig. S1 in the online Supplementary file, the product obtained after Li extraction from the spent LFP is identified as pure FePO_4_·2H_2_O, confirmed by X-ray diffraction (XRD). To monitor the real-time phase transition from FePO_4_·2H_2_O to NFPO, *in situ* variable-temperature XRD was performed. For clearer visualization, only the 10–34.3° region of the diffraction pattern is shown, while the complete spectra over the full angular range are provided (as shown in Fig. S2). As shown in Fig. 1, the phase and structural evolution from FePO_4_·2H_2_O to NFPO occurs in three distinct stages during continuous heating. The characteristic XRD peaks shift noticeably with increasing temperature, corresponding to the emergence and disappearance of different phases. In the first stage, FePO_4_·2H_2_O, which adopts the *Pnma* phase, remains stable below 200°C, although the intensity of its characteristic peaks diminishes as the temperature increases. In second stage, starting at 250°C, the XRD peaks begin to shift to lower angles, indicating the transformation of FePO_4_·2H_2_O phase. By 400°C, the FePO_4_·2H_2_O phase is nearly fully converted into the NaFePO_4_ phase, as evidenced by peak positions matching those of the olivine-structured NaFePO_4_ (PDF #97-016-9118). This phase transition marks the onset of Na intercalation. Simultaneously, the initial formation of NFPO can be detected. In the third stage, at around 450°C, the NaFePO_4_ phase begins to decompose, giving rise to the target NFPO product. The crystallinity of NFPO improves with further heating. Between 500°C and 550°C, PO_4_^3−^-based cathodes tend to decompose into P_2_O_7_^4−^-based products due to oxygen loss. This decomposition is driven by the reduced chemical potential of oxygen under a high-temperature, reducing atmosphere, which leads to oxygen evolution (O₂ release) and subsequent rearrangement of PO_4_^3−^ groups into P₂O₇⁴⁻ units [20]. During this third stage, the NFPO phase remains thermally stable in the 450–600°C range and persists throughout the 12-h heating period. From this real-time monitoring, a sequential phase transformation pathway, FePO_4_·2H_2_O → NaFePO_4_ → NFPO, can be clearly established. The structural evolution during heating is illustrated on the right side of Fig. 1, and the overall reaction pathway can be summarized as follows:


\begin{eqnarray*}
{\mathrm{FeP}}{{\mathrm{O}}}_4{\cdot} 2{\rm H}_2{\rm O} &+& {^{1}\!/\! _{2}} {\mathrm{N}}{{\mathrm{a}}}_4{\rm P}_2{\rm O}_6 \to {\mathrm{N}}{{\mathrm{a}}}_2{\mathrm{Fe}}{{\mathrm{P}}}_2{\rm O}_7\\
&+& 2{\rm H}_2{\rm O}
\end{eqnarray*}

The corresponding crystal structure evolution is depicted on the right side of Fig. 1. The NFPO framework adopts a triclinic *P*$\bar{1}$ phase and features a [Fe_2_O_11_] dimer, formed through edge-sharing and corner-sharing connections between [FeO_6_] octahedra and [P_2_O_7_] groups [21]. This arrangement constructs an open framework with spacious tunnels aligned along the [011] direction, which facilitates efficient Na^+^ ions migration [17]. Moreover, the robust structural backbone provides large interstitial voids to accommodate the reversible de-/intercalation of Na⁺ ions, suggesting the promising Na-storage capabilities of NFPO materials.

### Capture structure vacancy

**Figure 2.**
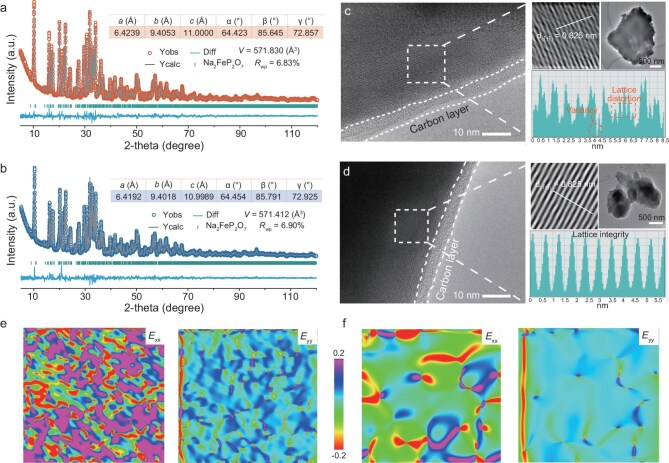
Structural vacancy characterization. Rietveld refinement results for XRD patterns of NFPO-R (a) and NFPO-D (b). HRTEM images of NFPO-R (c) and NFPO-D (d). In-plane (*E_xx_*) and out-of-plane (*E_yy_*) strain field maps obtained by GPA of NFPO-R (e) and NFPO-D (f).

Decades of research have identified Li^+^ ion loss and Fe^2+^ ion dissolution as the dominant factors contributing to LFP cathode degradation. These processes lead to the *in situ* formation of structural vacancy defects, specifically $V_{Li}^{\prime}$ and $V_{Fe}^{^{\prime\prime}}$ vacancies [22]. The presence of $V_{Li}^{\prime}$ facilitates the partial migration of Fe^2+^ ions into $V_{Li}^{\prime}$ sites, resulting in the formation of Li/Fe anti-site defects ($Fe_{Li}^ \cdot $), which hinder Li⁺ diffusion and further promote the generation of $V_{Fe}^{^{\prime\prime}}$ (Fig. S3) [9,22]. During the selective Li extraction process, the formation of $V_{Li}^{\prime}$ and $Fe_{Li}^ \cdot $ is effectively avoided, while $V_{Fe}^{^{\prime\prime}}$ are preserved in the FePO_4_·2H_2_O lattice. Based on this approach, we prepared NFPO materials using both spent LFP-derived FePO_4_·2H_2_O and a commercial counterpart, denoted as NFPO-R and NFPO-D, respectively. Rietveld refinement of the high-resolution powder XRD data using GSAS-II software (Fig. 2a and b) confirms that both NFPO-R and NFPO-D are single-phase materials, well-matched with the standard crystallographic pattern (PDF #01-080-2409). Notably, NFPO-R exhibits larger unit cell parameters than NFPO-D: *a *= 6.4239 Å, *b *= 9.4053 Å, *c *= 11.0000 Å, and volume (*V*) is 571.830 Å^3^ for NFPO-R, versus *a *= 6.4192 Å, *b *= 9.4018 Å, *c *= 10.9989 Å, and *V *= 571.412 Å^3^ for NFPO-D. The expanded unit cell of NFPO-R potentially enhances Na⁺ ion transport by widening migration channels and mitigating lattice strain during Na^+^ ion de-/intercalation. In addition, as detailed in Tables S1 and S2 in the online Supplementary file, the refined occupancy of Fe in NFPO-R is significantly lower than that in NFPO-D, consistent with the inductively coupled plasma optical emission spectroscopy (ICP-OES) results (Table S3), collectively confirming the presence of $V_{Fe}^{^{\prime\prime}}$ in NFPO-R. While electron paramagnetic resonance (EPR) is a sensitive technique for monitoring structural vacancies, Fe²⁺ ions possess an S = 0 antimagnetic ground state, rendering them ‘EPR silent’. As a result, $V_{Fe}^{^{\prime\prime}}$ cannot be directly observed via EPR. Nevertheless, their presence induces the formation of O vacancies ($V_O^{ \cdot \cdot }$), which produce a characteristic EPR signal at g = 2.003 (Fig. S4a and b), serving as indirect evidence of $V_{Fe}^{^{\prime\prime}}$. High resolution transmission electron microscopy (HRTEM) images (Fig. 2c and d) show that both NFPO-R and NFPO-D consist of micron-sized particles uniformly coated with a ∼3–4 nm carbon layer. While the two samples share similar particle sizes (1–2 μm), NFPO-R exhibits pronounced lattice distortion due to the presence of $V_{Fe}^{^{\prime\prime}}$, in contrast to the well-ordered lattice fringes in NFPO-D. The observed lattice disorder in NFPO-R arises from the altered coordination environments associated with $V_{Fe}^{^{\prime\prime}}$ formation. To investigate local strain behavior induced by $V_{Fe}^{^{\prime\prime}}$, geometrical phase analysis (GPA) was employed to map lattice distortions along the in-plane (*x*) and out-of-plane (*y*) directions. As shown in Fig. 2e and f, strain field maps (*E_xx_* and *E_yy_*) reveal more significant and uniformly distributed strain in NFPO-R compared to NFPO-D, suggesting that vacancy-induced internal stress is the primary driving force behind lattice distortion within NFPO-R.

### Vacancy-driven environmental alteration

**Figure 3.**
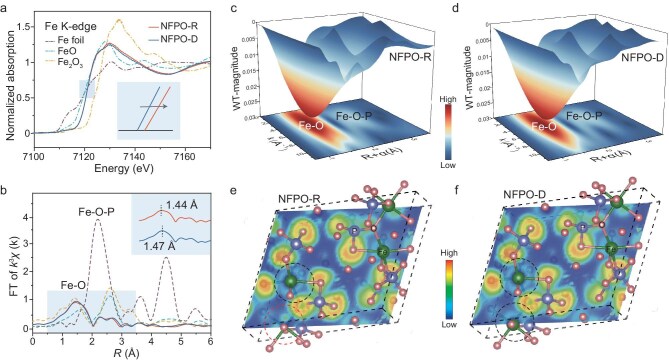
Electronic rearrangement by $V_{Fe}^{^{\prime\prime}}$. (a) Normalized Fe K-edge XANES spectra of the samples. (b) k^3^-weighted Fourier transform EXAFS spectra for the samples. WT for k^3^-weighted Fe K-edge EXAFS spectra for (c) NFPO-R and (d) NFPO-D. 2D ELF patterns and the crystal structures through (014) plane of (e) NFPO-R and (f) NFPO-D.

To investigate the differences in the local chemical environments of NFPO-R and NFPO-D, Fe K-edge X-ray absorption near-edge structure (XANES) spectra were conducted. As shown in Fig. 3a, both samples exhibit an Fe oxidation state of +2. However, due to variations in the coordination environment, the electron cloud density around Fe atoms differs. In NFPO-R, the XANES edge shifts to higher energy, which can be attributed to charge compensation triggered by $V_{Fe}^{^{\prime\prime}}$ defects, where Fe atoms contribute electrons to maintain charge neutrality [23]. The K-space spectra of NFPO-R display shorter oscillation periods and lower amplitudes compared to NFPO-D (Fig. S5), indicating a more distorted local structure and the presence of coordination defects associated with $V_{Fe}^{^{\prime\prime}}$. In the Fourier-transformed R-space of the Fe K-edge extended X-ray absorption fine structure (EXAFS) spectra (Fig. 3b), both samples show a prominent peak around 1.40 Å, corresponding to Fe–O bonds. The slight shift in the first coordination shell between NFPO-R and NFPO-D reflects minor variations in atomic distances [24]. EXAFS fitting results in Fig. S6 further confirm that the data for both samples can be well modeled using single scattering paths from the Fe–O first coordination shell. Additional insights are provided by wavelet-transformed (WT) EXAFS spectra (Fig. 3c and d), which reveal two dominant features at approximately 1.50 and 2.50 Å. These correspond to Fe–O and Fe–O–P interactions, respectively [25]. The presence of $V_{Fe}^{^{\prime\prime}}$ in NFPO-R leads to notable changes in its coordination environment, as reflected in reduced bond lengths of Fe–O–P interactions: 2.73 versus 2.85 Å for Fe1, and 3.26 versus 3.30 Å for Fe2, compared to NFPO-D. These shortened bond lengths suggest stronger bonding interactions, which contribute to enhanced structural stability in NFPO-R. Detailed fitting parameters are provided in Table S4. To further explore electronic structure changes, the electronic localization function (ELF) was calculated along the (014) plane for both materials. As shown in Fig. 3e and f, NFPO-D exhibits uniform charge distribution between Fe and O atoms (black circles), indicative of delocalized bonding. In contrast, NFPO-R demonstrates significant charge aggregation (red circles) around Fe–O bonds, a result of $V_{Fe}^{^{\prime\prime}}$ formation. The increased electron pair density in NFPO-R points to enhanced Fe–O bonding [26]. Taken together, these findings demonstrate that $V_{Fe}^{^{\prime\prime}}$ significantly alters the coordination environment, shortens bond lengths and increases local electron density in NFPO-R, potentially influencing its structural and electrochemical properties.

### Electrochemical properties

**Figure 4.**
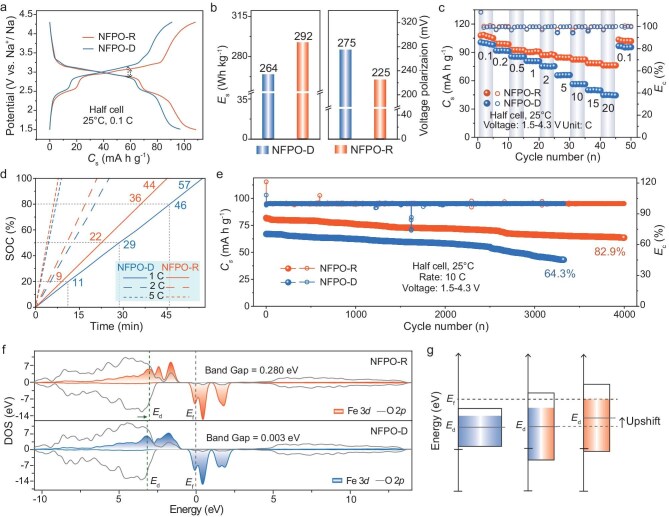
Electrochemical properties and electronic structure. (a) GCD curves at 0.1 C. (b) Es (left) and voltage polarization (right) comparison between NFPO-R and NFPO-D. (c) Rate capability. (d) SOC–time plots of the samples. (e) Cycling performance at 10 C. (f) pDOS plots of Fe 3*d* and O 2*p*. (g) Schematic representation of the elevated E_d_.

We further evaluate the electrochemical performance of the prepared Na-storage cathodes, as illustrated in Fig. 4. The galvanostatic charge–discharge (GCD) curves at 0.1 C reveal that both NFPO-R and NFPO-D exhibit multi-step potential-capacity profiles, attributed to the sequential occupation of distinct Na⁺ sites (Na1–Na6) with different local environments and orderings (Fig. 4a) [21]. In comparison, NFPO-R delivers a significantly higher specific capacity (*C_s_*) than NFPO-D, with more extended plateaus near 3 and 2.5 V (versus Na^+^/Na), reaching 107 versus 98 mAh g^−1^ for NFPO-D. These extended voltage plateaus also contribute to a higher average operating voltage for NFPO-R. As shown in Fig. S7, the middle voltage of NFPO-R remains higher than that of NFPO-D across various rates, with only a 0.31 V drop at 20 C relative to 0.1 C, compared to a 0.49 V drop for NFPO-D. As presented in Fig. 4b, the combination of extended voltage plateaus and increased capacity results in a higher specific energy density (E*_s_*) for NFPO-R (292.19 Wh kg^−1^) compared to NFPO-D (264.44 Wh kg^−1^). Moreover, NFPO-R shows a lower polarization (225 versus 275 mV), which is advantageous for electrochemical stability. Figure 4c demonstrates NFPO-R's excellent rate capability, as it fully recovers its original capacity upon returning from a high rate (20 C) to a low rate (0.1 C). Notably, NFPO-R retains 60% of its capacity at 20 C, while NFPO-D retains only 40%. The corresponding galvanostatic discharge curves are displayed in Fig. S8. As shown, NFPO-R maintains two apparent discharge plateaus even at high rate of 20 C, implying that $V_{Fe}^{^{\prime\prime}}$ confer high structural stability. In conventional Na-storage cathode design for fast-charging, introducing a vacancy-rich structure is a critical strategy to enhance fast-charging performance. This is because high-rate de-/sodiation during fast charging readily induces lattice contraction and distortion. Pre-engineered vacancies effectively buffer volumetric strain, maintain the structural stability of Na^+^ ion diffusion pathways, and thereby prevent Na^+^ ion transport blockage caused by structural collapse. To further assess fast-charging performance, we compared the time required to reach equivalent states of charge (SOC) at various C-rates (Fig. 4d). NFPO-R exhibits significantly improved charging kinetics, reaching 100% SOC at 1 C in 44 min, a 22.80% reduction in charging time compared to NFPO-D (57 min). At 5 C, NFPO-R requires only 1.46 min to reach 20% SOC and 6 min for 80% SOC, representing time savings of 23.56% and 17.01%, respectively, compared to NFPO-D. These enhancements are attributed to pre-vacancy engineering in NFPO-R, which induces self-adaptive lattice strain and forms a persistent, high-flux Na⁺ ions migration network. The corresponding galvanostatic charge curves are presented in Fig. S9. The long-term cycling stability was also evaluated over a voltage window of 1.5–4.3 V at 10 C. As shown in Fig. 4e, NFPO-R exhibits superior durability, retaining 82.9% of its capacity after 4000 cycles, while NFPO-D retains only 64.3% after just 3300 cycles. NFPO-R also maintains excellent high-temperature stability, achieving 600 cycles at 5 C and 60°C with nearly 100% Coulombic efficiency (*E_c_*) (Fig. S10). Electronic structure analysis (Figs 4f and S11) reveals that the Fe 3*d* partial density of states (pDOS) in both NFPO-R and NFPO-D crosses the Fermi level (E_f_), suggesting metal-like electronic conductivity. NFPO-R exhibits localized spikes in the pDOS, indicating enhanced electron localization, consistent with the ELF results in Fig. 3e. Moreover, the continuous bands near E_f_ further facilitate high electronic conductivity and efficient electron transport (Fig. S12). The calculated direct band gap between the valence band maximum (VBM) and conduction band minimum (CBM) is only ∼0.003 eV for NFPO-R, significantly narrower than 0.280 eV for NFPO-D (Fig. S13 and Tables S5–S6), indicating that $V_{Fe}^{^{\prime\prime}}$ greatly enhances the intrinsic conductivity of NFPO-R, contributing to its superior electrochemical behavior [11,27].

To explore the practical applicability of NFPO-R, a full cell configuration was assembled using NFPO-R as the cathode and a carefully screened hard carbon as the anode (Fig. S14). As shown in Fig. S15a, the full cell delivered a specific capacity of 101.7 mAh g^−1^ at 0.1 C, with a similar multi-step voltage profile as observed in the half-cell. The full cell also demonstrated excellent rate capability, with capacity recovery upon reverting from 20 to 0.1 C (Fig. S15b). Furthermore, the cell maintained 87.06% capacity retention after 200 cycles at 1 C, with nearly 100% Coulombic efficiency (Fig. S15c), underscoring strong potential of NFPO-R for real-world application. Additionally, pyrophosphate-based systems represent a structurally rich family of Na-storage cathode materials with diverse Na^+^ ion de-/intercalation behavior [28]. Importantly, degraded LFP cathode materials can be repurposed as feedstock for other Na–Fe–P–O series cathodes. As shown in Figs S16 and S17, cathode materials such as Na_3.12_Fe_2.44_(P_2_O_7_)_2_ and Na_3.32_Fe_2.34_(P_2_O_7_)_2_ were successfully prepared from precursors derived from spent LFP and exhibited excellent Na-storage performance. This demonstrates the versatility and scalability of vacancy-engineered pyrophosphate systems, offering a sustainable and cost-effective pathway for producing high-performance SIB cathodes from recycled LIB materials.

### Kinetics and Na-storage mechanism

**Figure 5.**
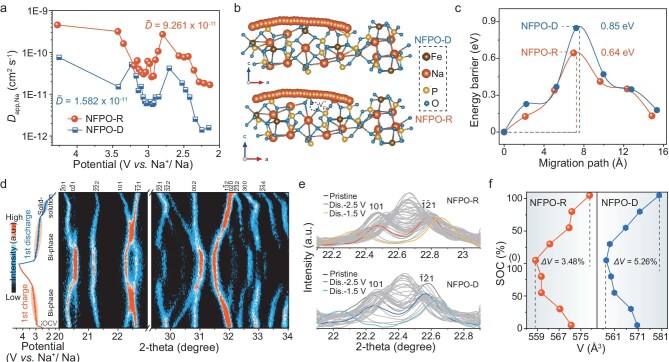
Electrode kinetics and Na-storage mechanism. (a) *D*_app, Na^+^_ calculated through GITT during discharge. (b) Diffusion path of NFPO-R and NFPO-D along (013) plane. (c) Calculated diffusion energy barrier profiles derived from (b). (d) 2D contour *in situ* XRD spectra of NFPO-R. (e) Stacked curves of the ($101$) and ($\bar{1}\bar{2}1$) plane during the first cycle. (f) Lattice *∆V* derived from *in situ* XRD results of (d).

In addition, the Na^+^ ion migration kinetics and Na-storage mechanisms were systematically investigated through both the experimental and theoretical approaches. As shown in Figs 5a and S18–S21, the kinetics diffusion coefficients (D_app, Na_) were derived from the galvanostatic intermittent titration technique (GITT) curves. Figure 5a illustrates that NFPO-R exhibits more stable D_app, Na_ values, with an average diffusion coefficient (${\mathrm{\bar{D}}}$_app, Na_) of 9.261 × 10^−11^ cm^2^ s^−1^, nearly an order of magnitude higher than that of NFPO-D (${\mathrm{\bar{D}}}$_app, Na_ = 1.582 × 10^−11^ cm^2^ s^−1^ for NFPO-D) during discharge. The corresponding diffusion energy barriers, presented in Fig. 5b and c, reveal that the presence of $V_{Fe}^{^{\prime\prime}}$ significantly lowers the Na^+^ ion migration barrier in NFPO-R (0.64 eV), compared to NFPO-D (0.85 eV), indicating enhanced carrier mobility. This improvement is attributed to the $V_{Fe}^{^{\prime\prime}}$-induced reduction in electrostatic repulsion among cations, which promotes the binding of larger-radius Na⁺ ions to anions. Additionally, the associated self-optimizing lattice distortion enhances Na⁺ ion diffusion kinetics, collectively contributing to the outstanding rate capability (Fig. 4c). To further elucidate the Na-storage behavior of NFPO-R and NFPO-D, *in situ* XRD measurements were conducted (Figs 5d and S22). During the initial charge–discharge cycle, a low-voltage plateau around 2.5 V was observed, corresponding to a solid-solution reaction characterized by peak shifts. In this stage, Na⁺ ions occupying the Na1 site are extracted along the [011] channel, with the XRD peaks shifting steadily and reversibly throughout cycling. Conversely, the high-voltage plateau centered at 3.0 V is associated with a biphasic reaction, marked by the emergence or disappearance of diffraction peaks. In this regimen, additional Na⁺ ions (Na3–Na6) are extracted via 1D and/or 2D diffusion pathways. The alternating solid-solution and biphasic reactions correlate with the SOC in the NFPO material. To further assess the impact of $V_{Fe}^{^{\prime\prime}}$ on structural stability, ($101$) and ($\overline {12} 1$) diffraction peaks are closely analyzed (Fig. 5e). During Na⁺ ion de-intercalation, both peaks undergo continuous phase transitions and shift back to their original positions upon re-intercalation, indicating a highly reversible Na⁺ ion de-/intercalation process. Notably, in NFPO-R, the peak positions at 2.5 V nearly coincide with those at open-circuit voltage (OCV) and shift gradually during discharge, suggesting excellent structural reversibility. In contrast, NFPO-D displays significant deviations in both peak position and intensity relative to OCV, reflecting incomplete Na⁺ ions intercalation. Lattice parameters were extracted from the *in situ* XRD data during Na⁺ ion de-/intercalation, and the variations in parameters *a*, *b* and *c* throughout the first cycle are presented in Fig. S23. As shown in Fig. 5f, NFPO-R experiences a smaller volume change (*∆V* = 3.48%) compared to NFPO-D (*∆V* = 5.26%). This reduced *∆V* suggests that structural vacancies in NFPO-R effectively accommodate the stress from repeated Na⁺ ion de-/intercalation, thereby enhancing long-term cycling stability.

### Techno-economic and life-cycle assessment

**Figure 6.**
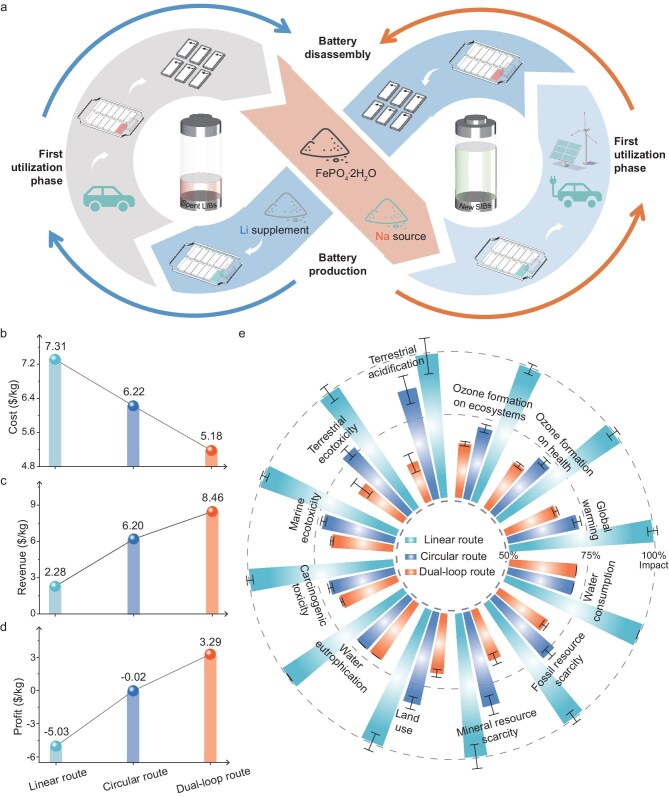
Techno-economic and life-cycle assessments. (a) Schematic illustration of the dual-loop route. Calculated 1 kg of products harvested by the three recycling methods in terms of cost input (b), revenue (c) and profit (d). (e) Environmental impacts comparison.

The successful implementation of a pre-engineered vacancy strategy for preparing the polyanionic Na-storage cathode materials has inspired us to develop a novel recycling approach for low-value, degraded cathode materials from LIBs. As shown in Fig. S24, Li is typically the only high-value mineral in LFP, making the profitability of recycling used LFP batteries largely dependent on Li recovery. However, Li only constitutes 4.4% of LFP, while the majority being low-value FePO_4_, resulting in limited economic returns that often fail to justify the high processing costs. Moreover, conventional hydrometallurgical recycling follows a linear ‘resource–product–waste’ model where waste generation is tolerated due to a primary focus on cost and recovery efficiency [6,29,30]. Unfortunately, this method relies heavily on hazardous chemicals and complex multi-step precipitation procedures, rendering it environmentally unsustainable [31–33]. To address both economic and environmental concerns, a circular recycling strategy has been explored [34,35]. This approach employs a mild Li extraction process, whereby recovered FePO_4_ is recombined with extracted Li_2_CO_3_ to remanufacture LFP cathodes, thus achieving a closed-loop cycle [10,36,37]. Nevertheless, this route still demands additional Li supplementation, which is susceptible to geopolitical tensions and resource scarcity, exposing it to potential supply chain disruptions [8]. To overcome these limitations and enhance the sustainable utilization of abundant resources, we propose a targeted recycling strategy. Following mild Li extraction from end-of-life LFP, the resulting FePO_4_ residue is combined with a cost-effective, abundant Na source to produce Na-storage cathode materials suitable for next-generation SIBs [38–41]. This hierarchical recycling of spent LFP cathodes enables the effective separation of high-value and low-value components, enabling their respective integration into distinct material circulation systems: LIBs and SIBs. This facilitates full life-cycle utilization and efficient management within each loop, constituting what we term the ‘dual-loop’ recycling pathway (Fig. 6a). Leveraging the intrinsic vacancy defects of the electrode materials, this dual-loop method transforms degraded materials into functional ones, marking a transformative shift from ‘failure’ to ‘functionality’.

We further assessed the economic viability and environmental impact of recycling 1 kg of LFP black mass using three different methods. Given that the residual Li content in spent LFP significantly influences the yield and quality of recovered products, we assumed a 90% residual Li content for all methods. The cost analysis accounted for ingredients, reagents, labor, water and electricity consumption, facility maintenance, and wastewater treatment, with specific values detailed in Table S7. As shown in Fig. 6b, c and d, the linear recycling method incurs high reagent costs and yields minimal economic return, resulting in a net loss of $5.03/kg. The circular method offers better reagent safety margins and a lower base cost ($6.22/kg), but its reliance on costly Li_2_CO_3_ supplementation leads to significant cost variability and a marginal net loss (−$0.02/kg). In contrast, the dual-loop method eliminates the need for Li supplementation by using low-cost Na, reducing the cost to $5.18/kg. Furthermore, this approach generates substantial revenue from both Li_2_CO_3_ and moderately valued NFPO, achieving the highest net profit of $3.29/kg.

Environmental impact is another crucial metric in evaluating the feasibility of recycling approaches. Using the ReCiPe 2016 Midpoint methodology within the OpenLCA framework, we conducted a comprehensive life-cycle assessment (Tables S8–S10). As shown in Fig. 6e and Table S11, the dual-loop approach demonstrates superior environmental performance across key categories, including resource scarcity, human health and ecosystem quality. For example, the dual-loop route reduces global warming potential by 30.236% compared to the linear route and by 9.741% relative to the circular route. In terms of fossil resource scarcity, it shows a 29.102% reduction over the linear route and 12.609% over the circular route.

In conclusion, techno-economic and life-cycle assessments affirm that the dual-loop recycling strategy offers significant advantages in terms of cost-effectiveness, environmental impact and resource sustainability. By bridging the gap between end-of-life LIBs and emerging SIB technologies, this dual-loop design presents a compelling solution for the sustainable recycling of low-value cathode materials.

## CONCLUSIONS

In summary, a self-adaptive pre-vacancy engineering strategy was developed to enhance the fast-charging capabilities of Na–Fe–P–O cathode materials for SIBs. Unlike traditional methods that rely on artificially introducing defects, this strategy leverages the naturally occurring $V_{Fe}^{^{\prime\prime}}$, which are typically viewed as detrimental in spent LiFePO_4_ cathodes, and repurposes them as functional components in Na-storage materials. Compared to the defect-engineered NFPO-D, the electrochemically evolved $V_{Fe}^{^{\prime\prime}}$ in NFPO-R introduces additional Na-storage sites, effectively mitigating volumetric strain during repeated Na⁺ ion de-/intercalation cycles. Moreover, these vacancies induce localized electron clustering, which lowers the activation energy and facilitates improved charge transfer kinetics. This innovative ‘failure-to-functionality’ approach has demonstrated outstanding electrochemical performance across various Na–Fe–P–O cathode materials, confirming its broad applicability. Building on this success, we propose a comprehensive dual-loop recycling paradigm that extends the impact of pre-vacancy engineering by linking end-of-life LIBs with next-generation battery technologies such as SIBs. The dual-loop recycling strategy not only delivers significant environmental and economic benefits but also presents a transformative solution for the sustainable management of low-value spent LIBs, while simultaneously accelerating advancements in secondary battery systems.

## Supplementary Material

nwaf321_Supplementary_Data
