# Supplementary material for: Dual-loop upcycling of spent LiFePO4: defect inheritance enables durable and fast-charging sodium-ion batteries
Source: Natl Sci Rev. 2025 Aug 8;12(9):nwaf321. doi: 10.1093/nsr/nwaf321 (PMC12421578; doi:10.1093/nsr/nwaf321)
Supplement: nwaf321_Supplementary_Data [file nwaf321_supplementary_data.pdf]

Supplementary Data for

Dual-loop upcycling of spent $LiFePO_4$: defect inheritance enables durable and fast-charging sodium-ion batteries

Xiao-Tong Wang,[1] Zhen-Yi Gu,[1] Jun-Ming Cao,[1] Xin-Xin Zhao,[2] Han-Hao Liu,[2] Shuo-Hang Zheng,[1] Yong-Li Heng,[1] Kai-Yang Zhang,[1] Edison Huixiang Ang,[3] Zhe Wang,[4] Ronghua Zeng[4] and Xing-Long Wu[1,2,]*

[1]State Key Laboratory of Integrated Optoelectronics, MOE Key Laboratory for UV Light-Emitting Materials and Technology, Northeast Normal University, Changchun 130024, P. R. China

[2]Department of Chemistry, Northeast Normal University, Changchun 130024, P. R. China

[3]Natural Sciences and Science Education, National Institute of Education, Nanyang Technological University, Singapore 637616, Singapore

[4]Guangdong Provincial International Joint Research Center for Energy Storage Materials, School of Chemistry, South China Normal University, Guangzhou 510006, P. R. China

***Corresponding author.** Email: xinglong@nenu.edu.cn

Experimental Section/Methods

Access to reagents and raw materials. The chemical reagents used included sodium chloride (NaCl, A.R., 99%, Sinopharm Chemical Reagent), sodium persulfate ($Na_2S_2O_8$, A.R., 99%, Adamas), sodium carbonate (Na_2CO_3, A.R., 99%, Tianjin Guangfu Chemical Reagent), sodium pyrophosphate ($Na_4P_2O_7$, A.R., 99%, Macklin), citric acid ($C_6H_8O_7$, A.R., 99%, Macklin). The spent LFP batteries were supplied by a local battery company. After soaking discharge in 1M NaCl solution for 24h and manual disassembly under safe conditions, the failed LFP electrode sheets were obtained. The obtained LFP electrode sheets were subsequently sintered in a tube furnace filled with Ar atmosphere at 550 ℃ for 4 h. Due to the failure of the binder at high temperatures, the active materials were easily peeled off from Al foil, resulting in a plentiful of LFP powder.

Dual-loop recycling process. The spent LFP powder and $Na_2S_2O_8$ were mixed in the molar ratio and then a certain amount of distilled water was added to achieve a solid-liquid ratio of 5% (w/v). After stirring for a certain time, filtration was carried out to obtain the separated solid $FePO_4 \cdot 2H_2O$ and Li-rich solution. The latter was precipitated by Na_2CO_3 to obtain Li_2CO_3. The selective chemical delithiation can be written as:

$$Na_2S_2O_8 + 2Li_{(1-x)}FePO_4 = 2FePO_4 + (1-x)Li_2SO_4 + Na_2SO_4.$$

$$Li_2SO_4 + Na_2CO_3 = Li_2CO_3\downarrow + Na_2SO_4.$$

Where x is the molar amount of residual Li in the spent LFP cathode materials.

The dried $FePO_4 \cdot 2H_2O$ was placed in agate jars with $Na_4P_2O_7$ and $C_6H_8O_7$ according to stoichiometric ratio and wet ball milling was carried out with ethanol as dispersant for 12 h. After removal of excess ethanol by rotary evaporation and drying, the collected samples were sintered in a tube furnace in a mixed gas atmosphere (95% Ar + 5% H_2) at 600 ℃ for 12 h to obtain NFPO-R samples. NFPO-D was synthesized in the same steps as above, except that the raw material was commercial $FePO_4 \cdot 2H_2O$.

Materials characterization. The chemical composition of NFPO samples was determined by ICP-OES (Agilent ICPOES730, USA). Crystalline phase analyses were evaluated by X-ray diffraction (XRD) patterns, which were collected on a Rigaku SmartLab X-ray diffractometer (Cu-Kα radiation, λ = 1.5418 Å, current: 30mA, voltage: 40 kV) in the scan range (2θ) of 5°- 80°. The particle size and morphology of the materials were investigated by using the scanning electron microscopy (SEM, Hitachi SU 8000). A transmission electron microscope (TEM, JEQL-2100F) was used to capture the morphology of NFPO materials. Structural changes were monitored by in situ variable temperature (Germany Bruker D8 Advance X-ray diffractometer). Electron Paramagnetic resonance (EPR, Bruker A300-10/12, Germany) was used to detect the type and intensity of defects in the sample. X-ray absorption fine structure (XAFS) spectroscopy was carried out using the *RapidXAFS* 2M (Anhui Absorption Spectroscopy Analysis Instrument Co., Ltd.) by transmission mode at 20 kV and 20 mA, and the Si (531) spherically bent crystal analyzer with a radius of curvature of 500 mm was used for Fe.

Electrochemical performance tests. The cathode was made from a slurry consisting of 80wt% active material, 10wt% Ketjen black (KB, Canrd Technology Co. Ltd) and 10wt% sodium alginate (SA, Aladdin) coated on carbon-coated Al foil. The anode was made from a slurry consisting of 70wt% HC active material, 20wt% conductive carbon (C45, Canrd Technology Co. Ltd) and 10wt% carboxyl methyl cellulose (CMC, DoDoChem) coated on Al foil. Immediately after oven drying them were transferred to a glove box filled with Ar atmosphere for the assembly of 2032-type coin cell. The electrolyte was 1 mol·L^{-1} $NaClO_4$ in propylene carbonate (PC) with 10 vol% fluoroethylene carbonate (FEC). The glass fiber membranes were employed as the separator, and metallic Na was used as both counter and reference electrodes in the half cells. The galvanostatic charge-discharge (GCD) measurements were conducted on the battery testing systems (Neware) in the potential window of 1.5-4.3 V vs. Na^+/Na at different rates. The galvanostatic intermittent titration technique (GITT, Arbin battery tester, USA) were used to study the electrode kinetic via calculating the apparent Na^+ diffusion coefficients ($D_{app,Na}$). Prior to full cell assembly, electrodes were paired based on an anode-to-cathode capacity ratio (NP = 1.05-1.2). The anode pre-sodiation procedure involved depositing 100 μL electrolyte onto sodium metal, followed by pressing the metal onto HC anode to ensure intimate contact, with subsequent resting for 3-7 hours. The pre-sodiated anodes and matched cathodes were assembled into 2032-type coin cells using identical electrolyte formulation (1 mol·L^{-1} $NaClO_4$ in PC with 10vol% FEC). The C_s of the full cell were calculated with reference to cathode active material mass within a 1.2–4.3 V (vs. Na^+/Na) voltage window.

Theoretical calculation. All the density functional theory (DFT) calculations were carried out using the Vienna Ab initio Simulation Package (VASP) [1,2]. The exchange–correlation effects were handled using the generalized gradient approximation (GGA) of the Perdew-Burke-Ernzerhof (PBE) [3,4]. The interactions between core and valence electrons were described by the projector augmented wave (PAW) method [5]. The structure relaxations were performed with 480 eV plane-wave cutoff energy. The convergence criteria of supercell optimization for energy and force were set as 10^{-5} eV and 0.02 eV $Å^{-1}$, respectively. And the energy barriers for Na^+ ion diffusion in the structure were computed by the nudged elastic band (CI-NEB) method with 5 images.

Techno-economic and life-cycle assessments. In this study, the environmental footprints of three spent LFP battery recycling technology routes were systematically constructed and compared using the full life cycle assessment method. The study sets a clear system boundary to cover the complete process of spent LFP battery recycling, and takes the recycling and treatment of 1kg of spent LFP black mass as a functional unit to achieve a standardized comparison of the environmental footprints of the three different routes. The study builds an assessment model based on OpenLCA software and selects the ReCiPe 2016 Midpoint method for life cycle impact evaluation. The evaluation system integrates international mainstream evaluation methods such as CML, Eco-indicator and TRACI, and comprehensively covers the three major

protection areas of human health, ecosystem quality and resource consumption through 18 midpoint indicators, which ensures the systematicity and completeness of the evaluation. All the characterization models have been widely validated by the international LCA academic community, ensuring the scientific and comparable results of the study. In order to deal with the uncertainties in the study, the following assumptions are set in this study: (1) Auxiliary factors such as production equipment, plants, personnel and transport vehicles are not taken into account in the production stage; (2) For the material and energy flows, the upstream production process of a unit process that is less than 5 wt% by weight and does not contain rare or high purity components (less than 0.1 wt% when it does) can be ignored, but the total amount of the ignored amount is not more than 5 wt%. These assumptions effectively control the uncertainty in the research process and ensure the reliability of the assessment results.

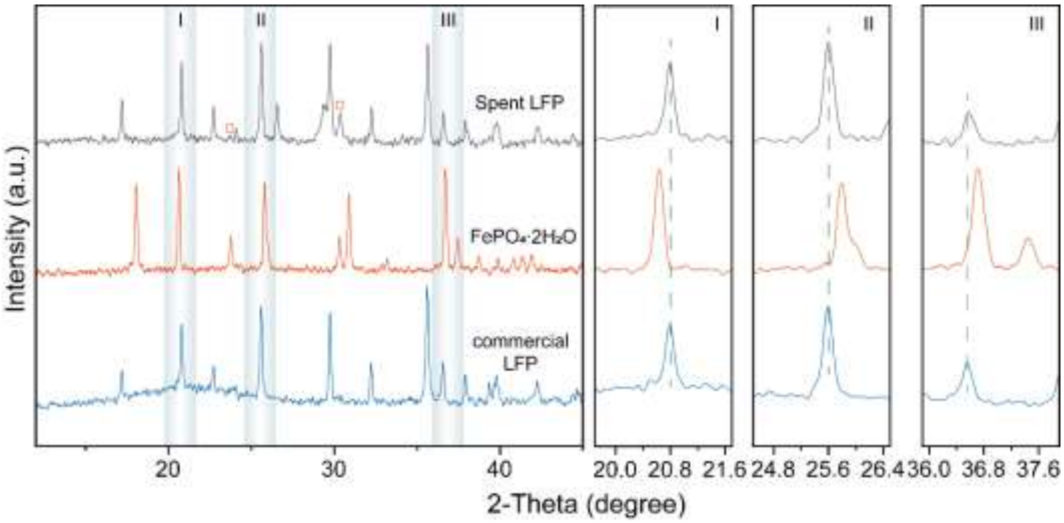

Figure S1. Comparison of XRD patterns among spent LFP, $FePO_4{\cdot}2H_2O$ and commercial LFP, with the right images being partial enlargements of the left one. As can be seen, the spent LFP cathode material is in the state of coexistence of two phases, LFP and $FePO_4$. After Li extraction, the harvested $FePO_4{\cdot}2H_2O$ exhibits completely different diffraction peak positions and intensities from commercial LFP.

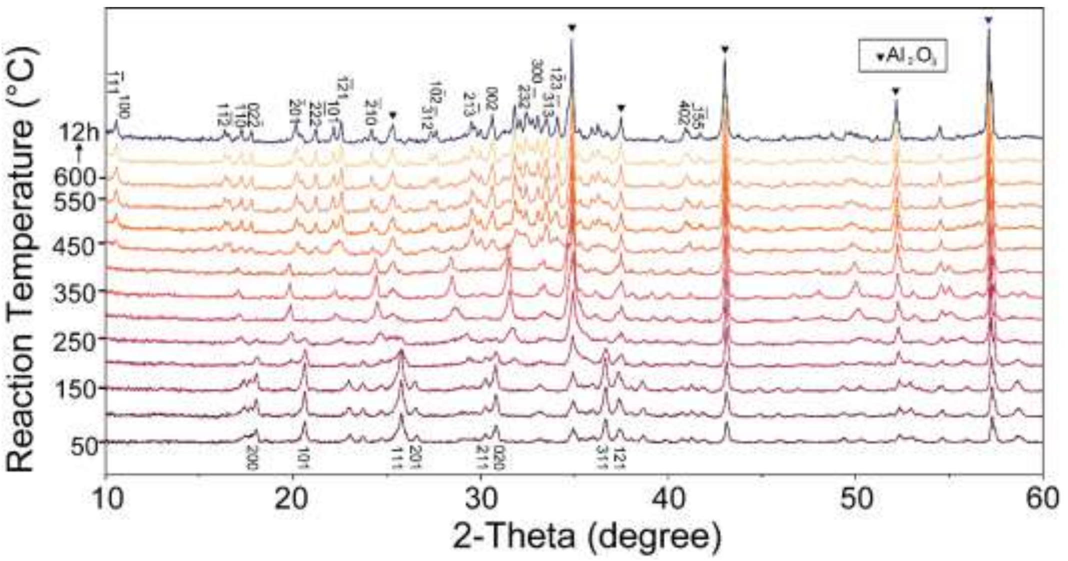

Figure S2. Full variable-temperature XRD range line spectra, where the peaks falling at 25.499 °, 35.056 °, 37.714 °, 43.298 °, 52.467 °, and 57.425 ° refer to the Al2O3 black specimen.

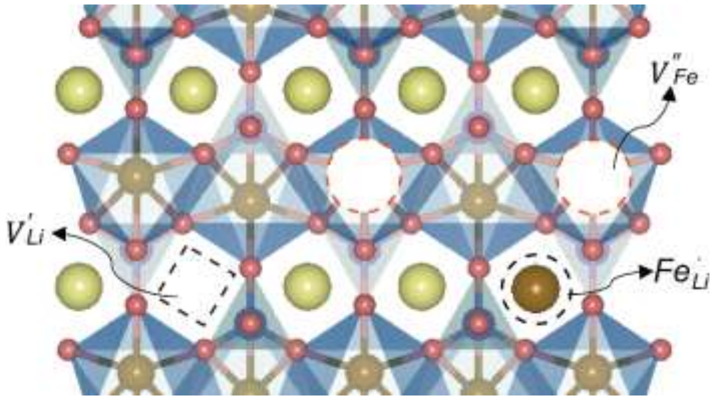

Figure S3. Failure mechanism of spent LFP cathode materials.

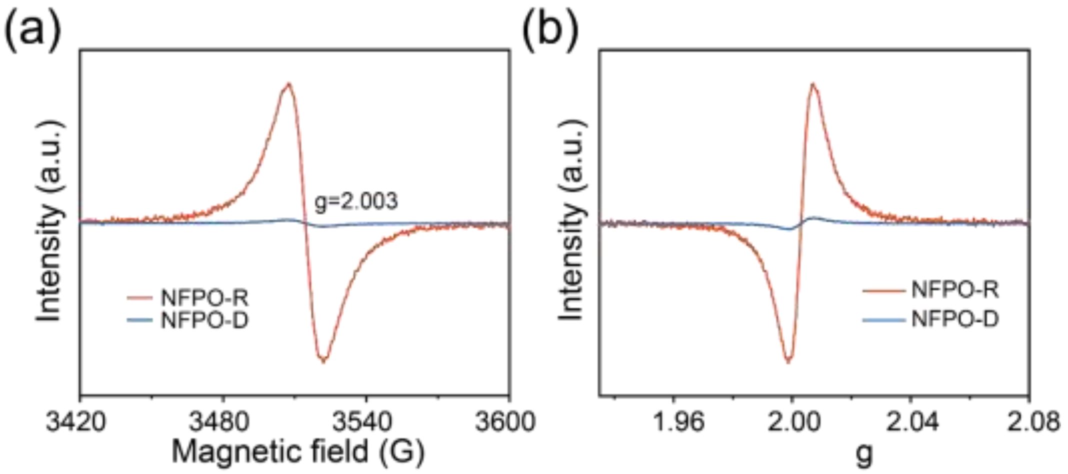

Figure S4. (a) EPR spectra of NFPO-R and NFPO-D. (b) g factor of NFPO-R and NFPO-D.

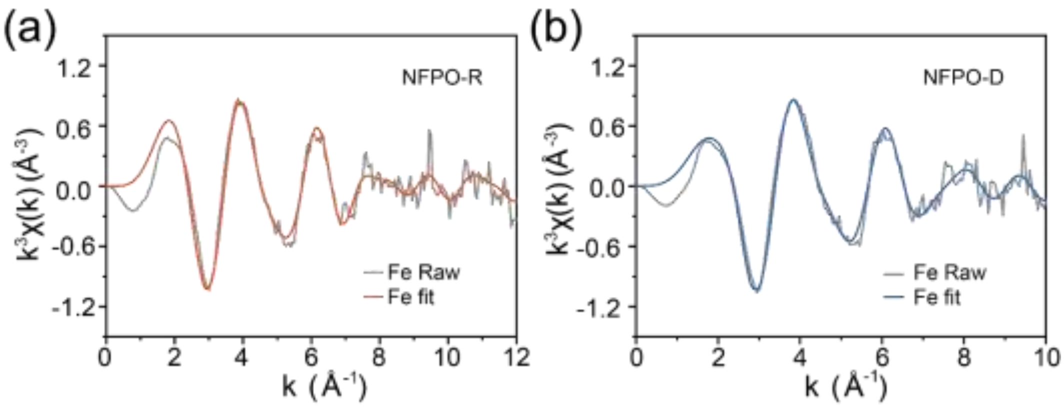

Figure S5. Fe *K*-edge oscillation functions for (a) NFPO-R and (b) NFPO-D.

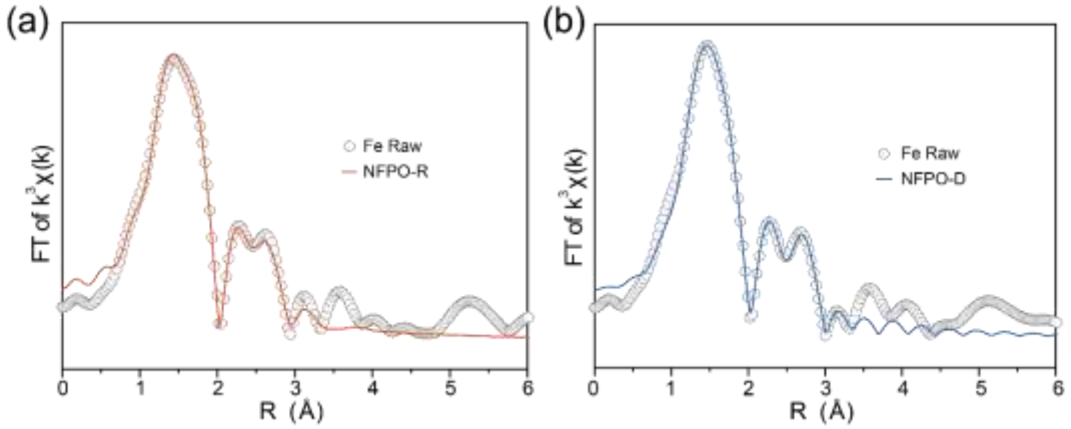

Figure S6. FT-EXAFS fitting curves at R space for (a) NFPO-R and (b) NFPO-D.

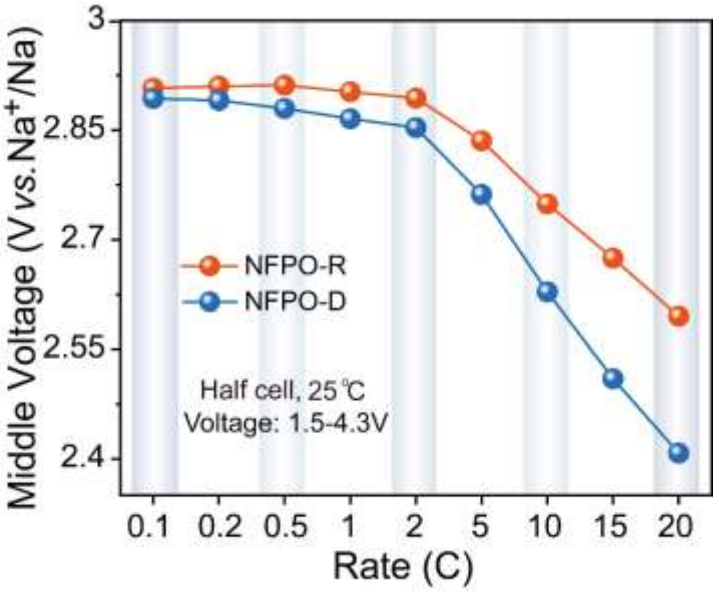

Figure S7. Middle voltage comparison between NFPO-R and NFPO-D.

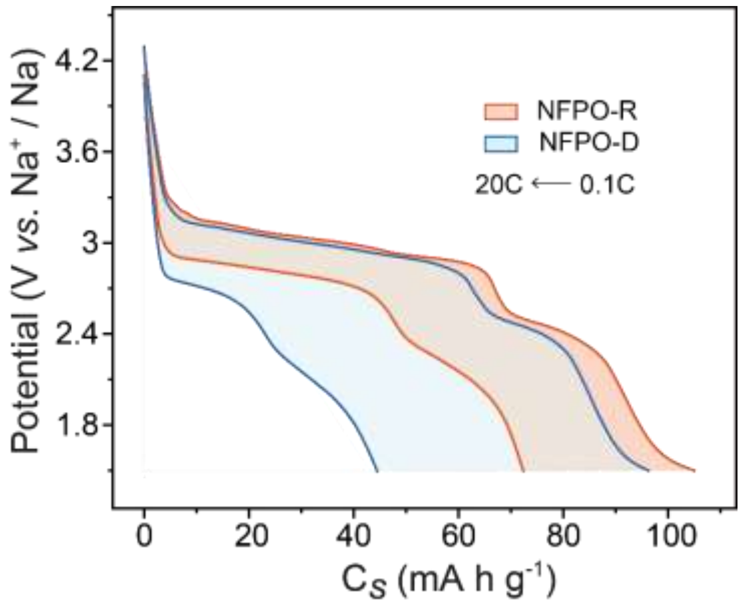

Figure S8. Galvanostatic discharge curves at various rates.

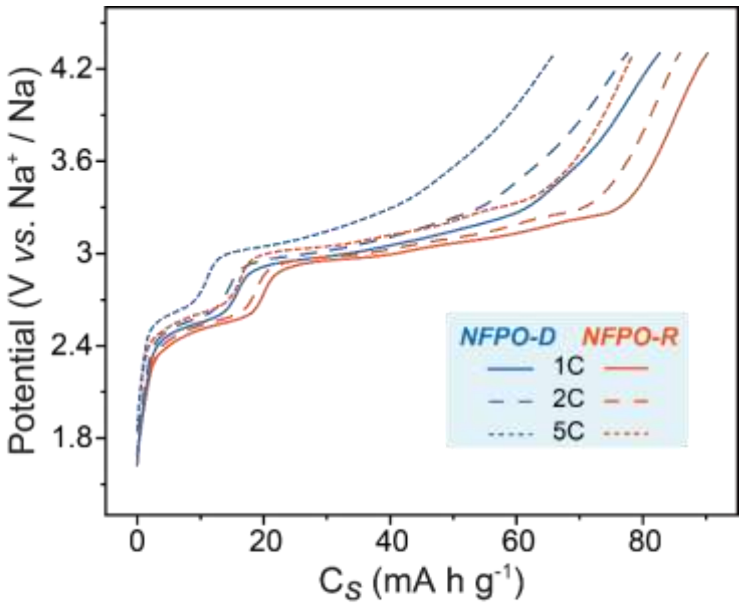

Figure S9. Galvanostatic charge curves in fast-charging scenario.

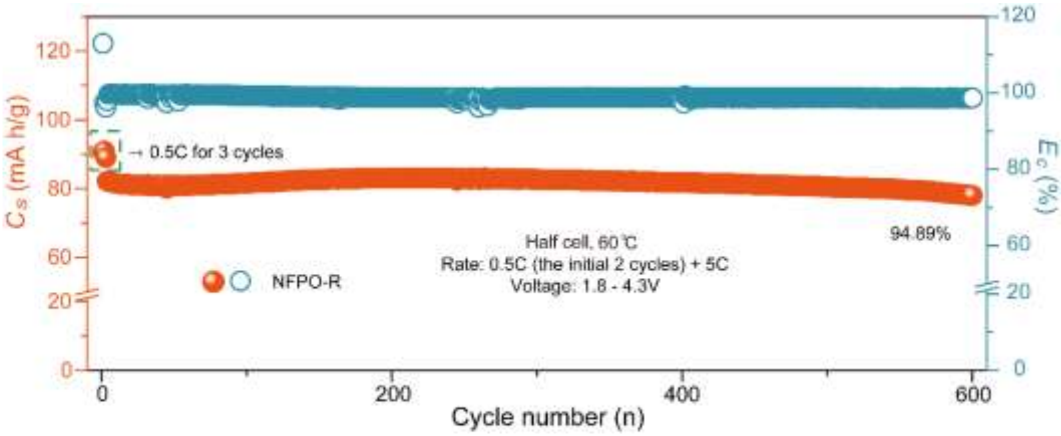

Figure S10. Cycling performance of NFPO-R cathode materials at high temperature. The half cell first underwent three cycles of activation at 0.5C, followed by 600 cycles at high rate of 5C, with 94.89% capacity retention.

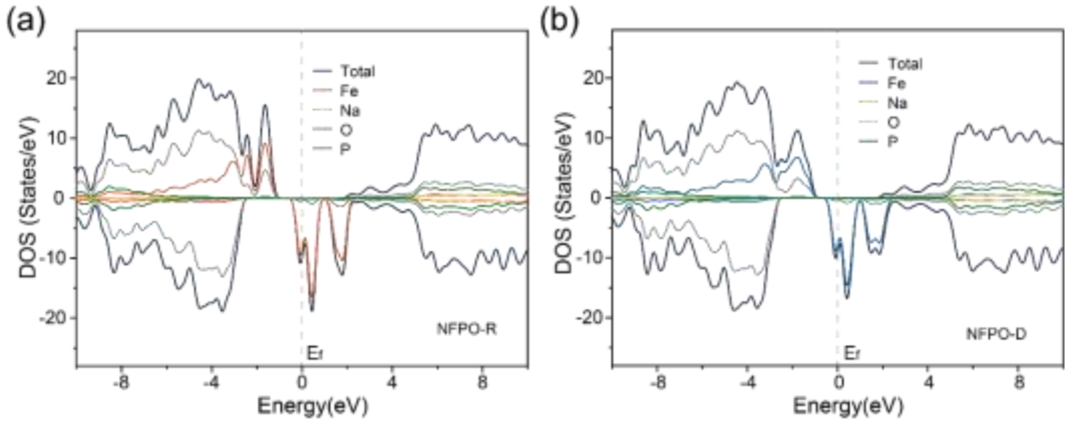

Figure S11. Total density of states and pDOS diagram of NFPO-R (a) and NFPO-D (b).

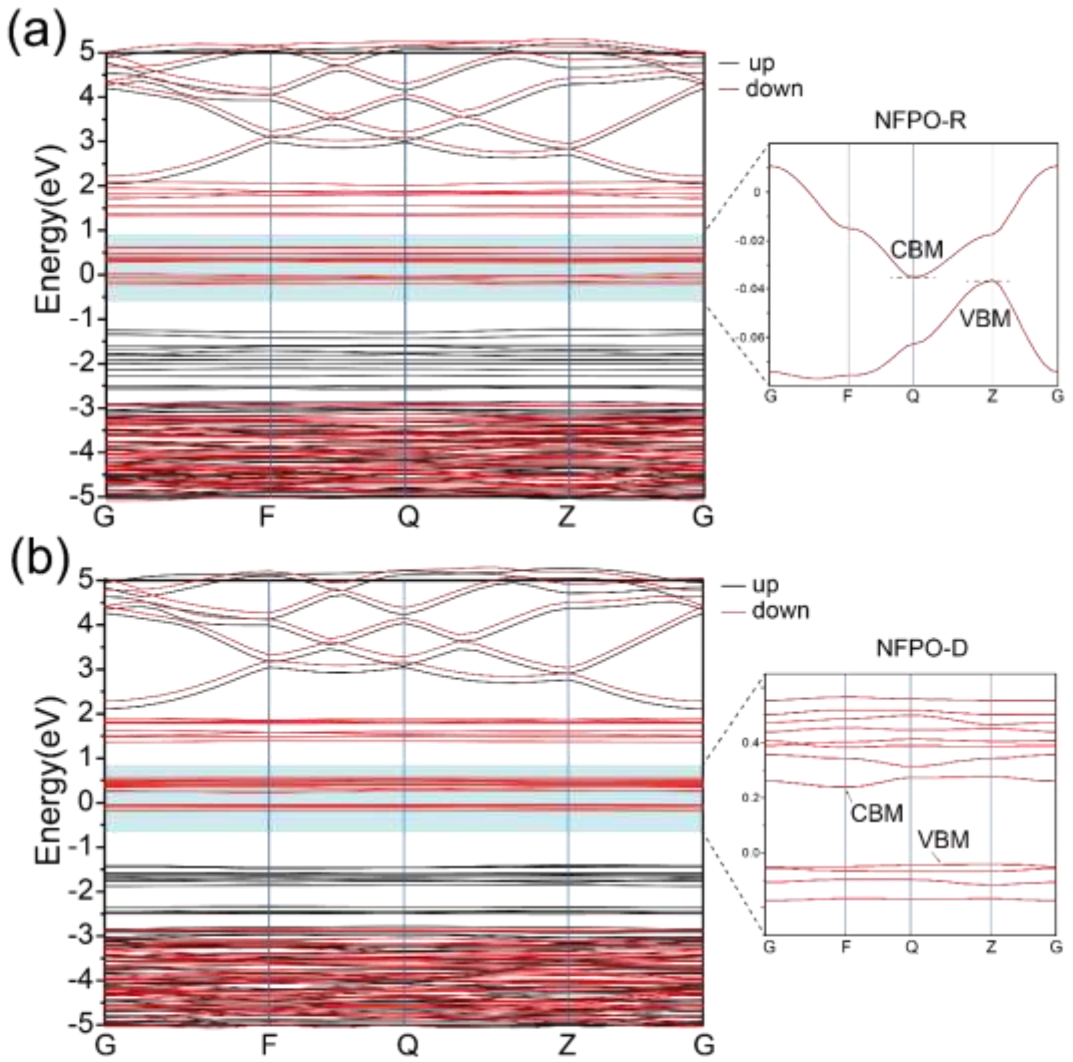

Figure S12. Electric band structure diagram of NFPO-R (a) and NFPO-D (b).

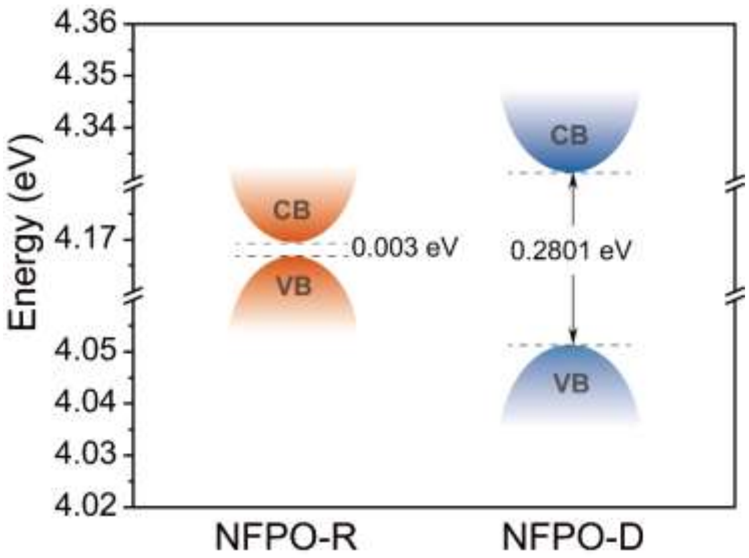

Figure S13. Schematic illustration of band gaps of NFPO-R and NFPO-D.

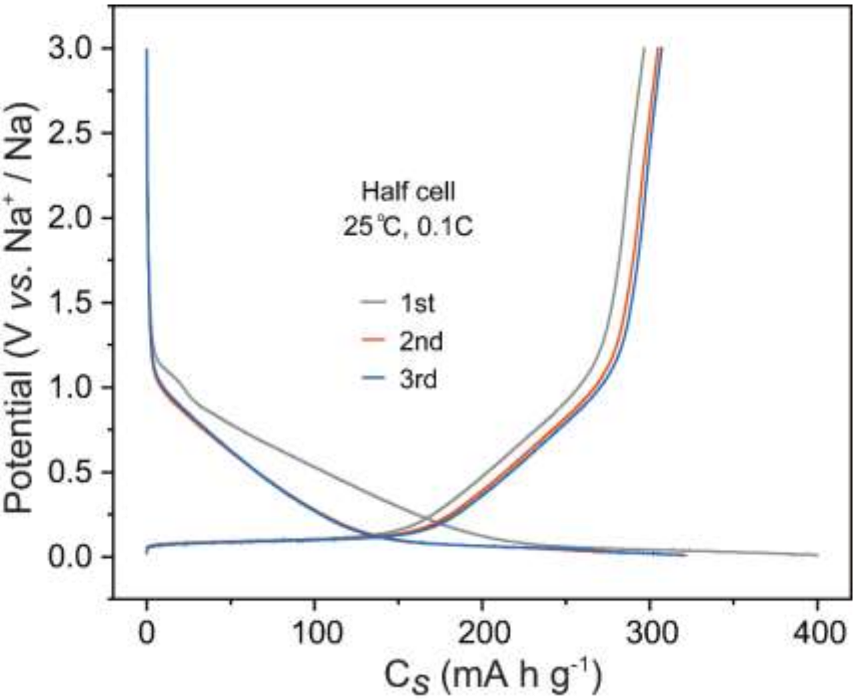

Figure S14. GCD curves for the first 3 cycles of HC anode.

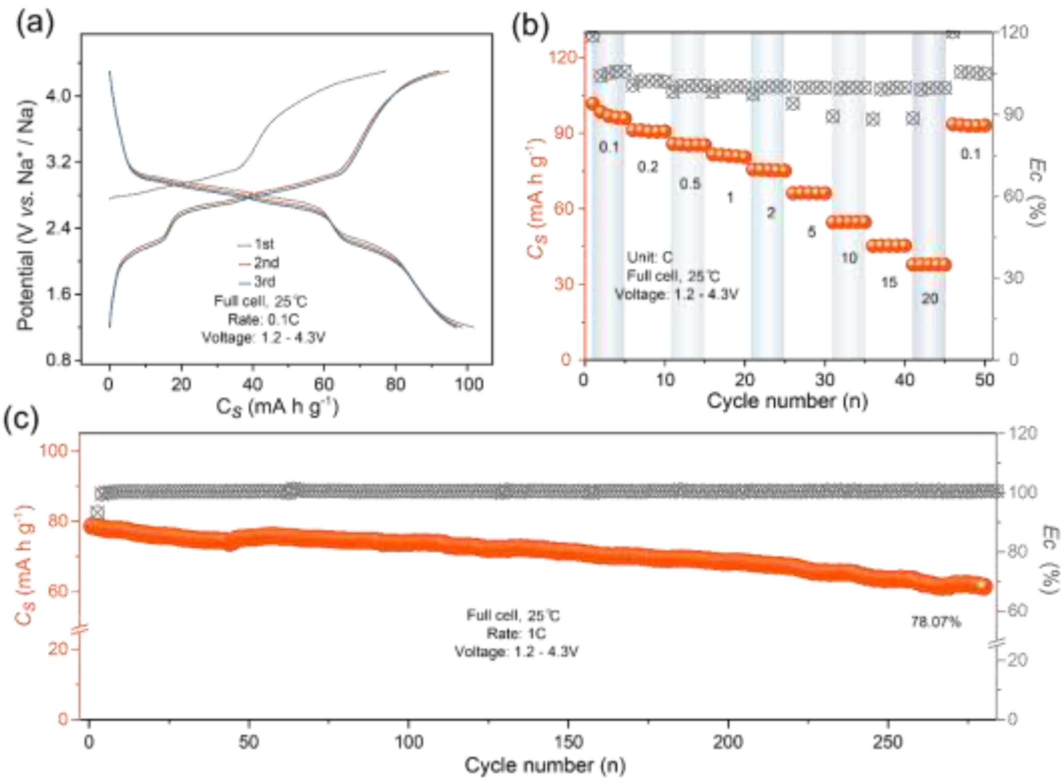

Figure S15. Electrochemical performance of NFPO-R // HC full cell. (a) GCD curves for the first 3 cycles at 0.1C. (b) Rate capability (c) Cycle performance at 1C.

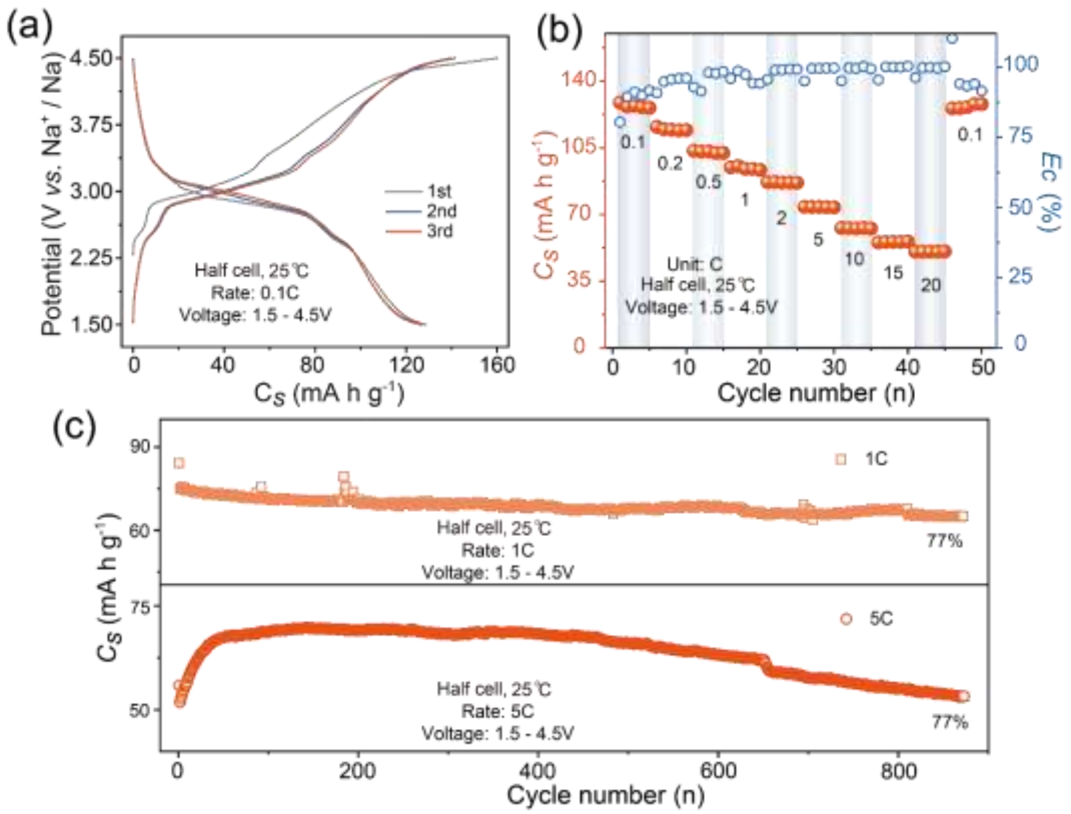

Figure S16. Electrochemical performances of $Na_{3.12}Fe_{2.44}(P_2O_7)_2$ in half cell. (a) GCD curves for the first 3 cycles at 0.1C. (b) Rate capability and (c) cycle performance at 1C and 5C.

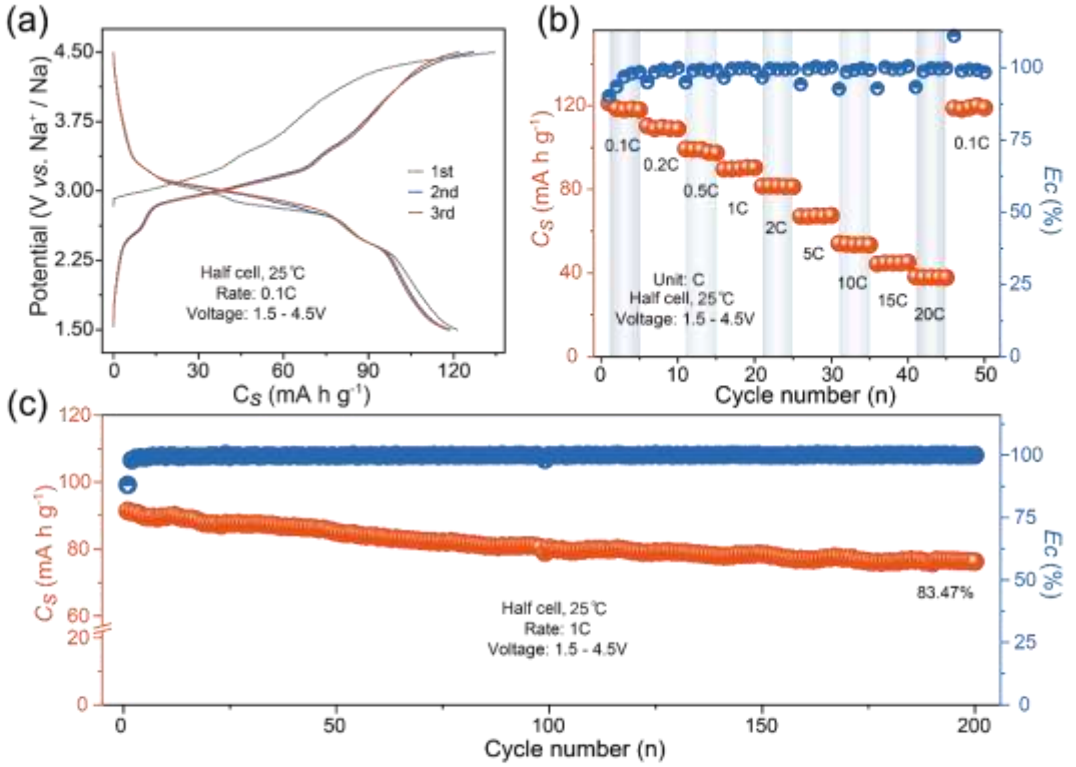

Figure S17. Electrochemical performances of $Na_{3.32}Fe_{2.34}(P_2O_7)_2$ in half cell. (a) GCD curves for the first 3 cycles at 0.1C. (b) Rate capability and (c) cycle performance at 1C.

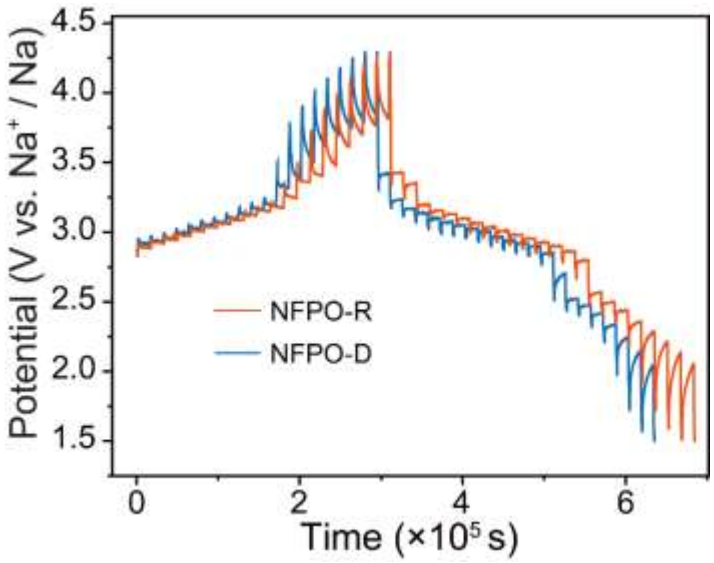

Figure S18. Typical GITT curves of NFPO-R and NFPO-D.

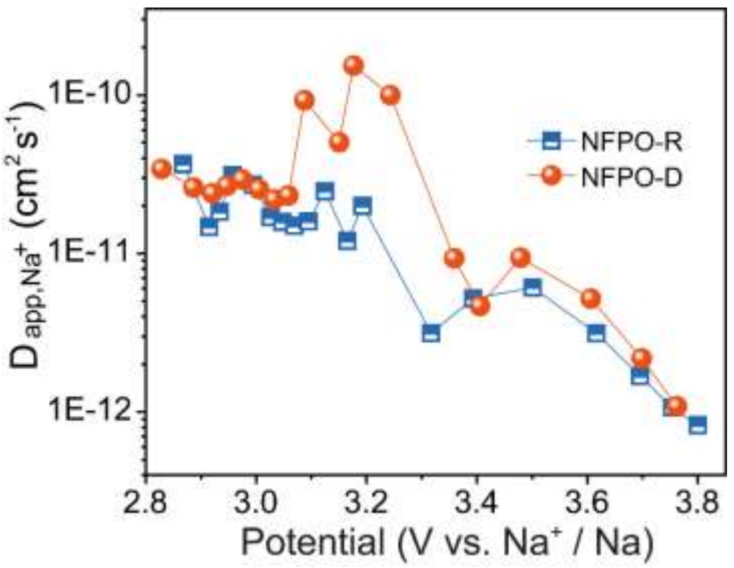

Figure S19. Calculated diffusion coefficients of NFPO-R and NFPO-D during charging.

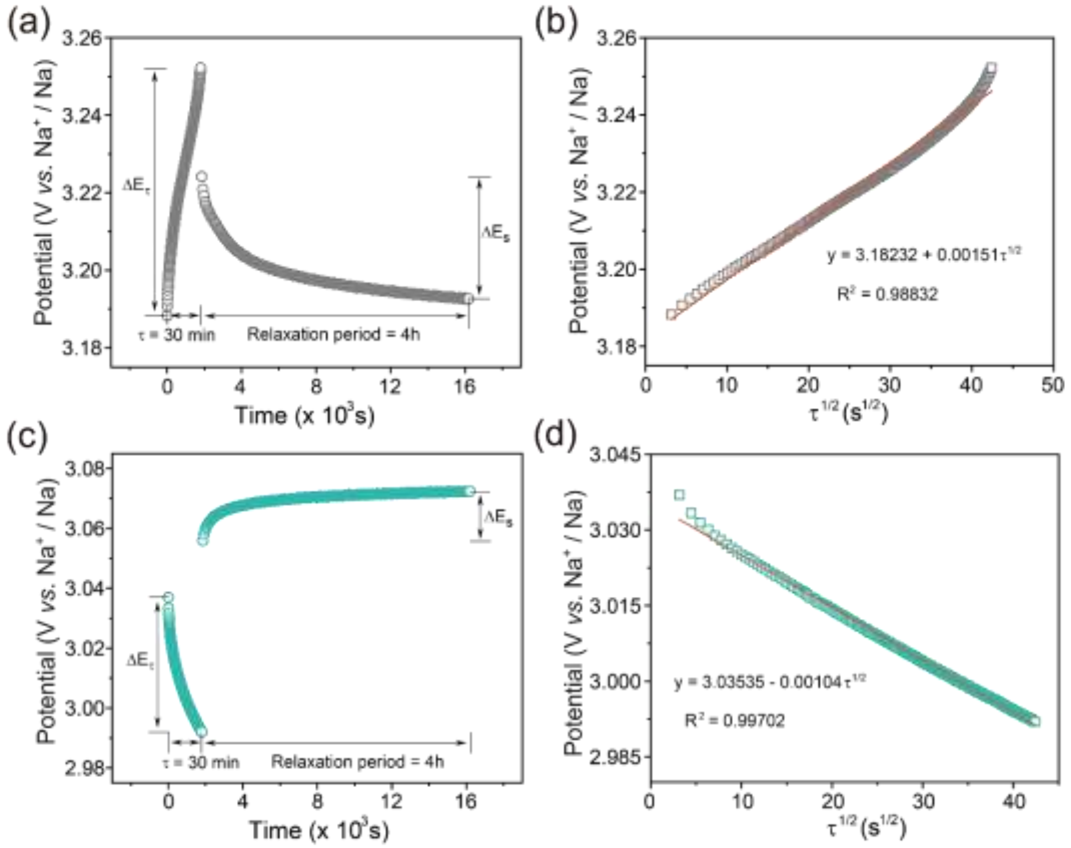

Figure S20. Representative individual galvanostatic titration procedure for NFPO-R during (a) charging and (c) discharging process, and the corresponding linear fitting behavior of E *vs.* $\tau^{1/2}$ in (b) charging and (d) discharging process.

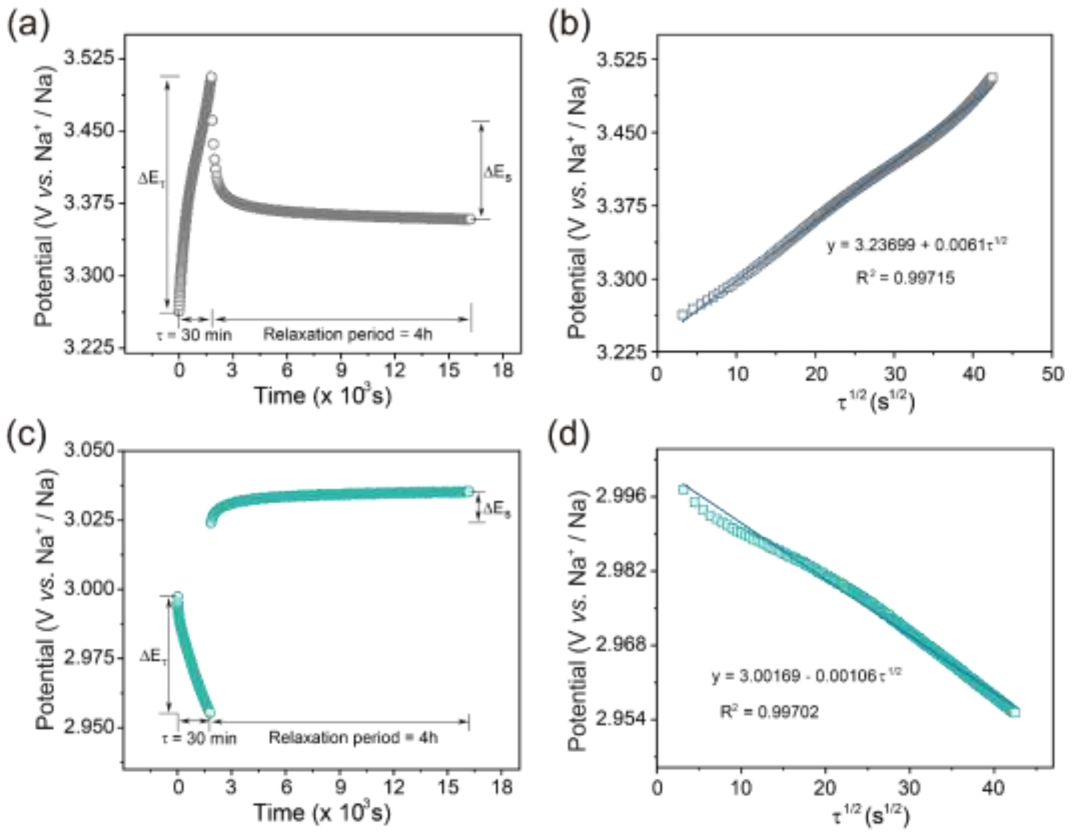

Figure S21. Representative individual galvanostatic titration procedure for NFPO-D during (a) charging and (c) discharging process, and the corresponding linear fitting behavior of E *vs.* $\tau^{1/2}$ in (b) charging and (d) discharging process.

The $D_{app,Na}$ values of NFPO-R and NFPO-D cathode materials were calculated by measuring GITT, according to Fick's second law. During the tests, the representative GITT curves were tested under the cycling conditions of 0.05C, where the duration time (τ) of each current pulse was set to 1800 s with a relaxation time of 14400 s after each pulse.

During charging process, the potential first rises rapidly, which can be attributed to the electrical internal resistance of the electrodes. Subsequently, the rise in potential slows down, which is due to the electrochemical Na^+ intercalation upon galvanostatic charging. After each current pulse, the potential rises momentarily due to the electrical internal resistance. Finally, the potential gradually reaches the quasi-equilibrium open-circuit potential. The discharging process is reversed.

The $D_{app,Na}$ values can be calculated as equation [6,7]:

$$\boldsymbol{D_{app,Na}} = \frac{\boldsymbol{4}}{\boldsymbol{\pi\tau}}\left(\frac{\boldsymbol{m_B V_M}}{\boldsymbol{M_B S}}\right)2\left(\frac{\boldsymbol{\Delta E_s}}{\boldsymbol{\Delta E_\tau}}\right)2 \qquad (\boldsymbol{\tau \ll L^2/D})$$

where m_B, M_B, and V_M are the mass, molecular weight, and molar volume of the NVPOF material, respectively; τ is the duration of the current pulse; S is the apparent area of the electrode surface; L is the average radius of the material particles; and ΔE_s is the potential change in a single-step of current pulse; ΔE_τ is the quasi-equilibrium potential change between steps.

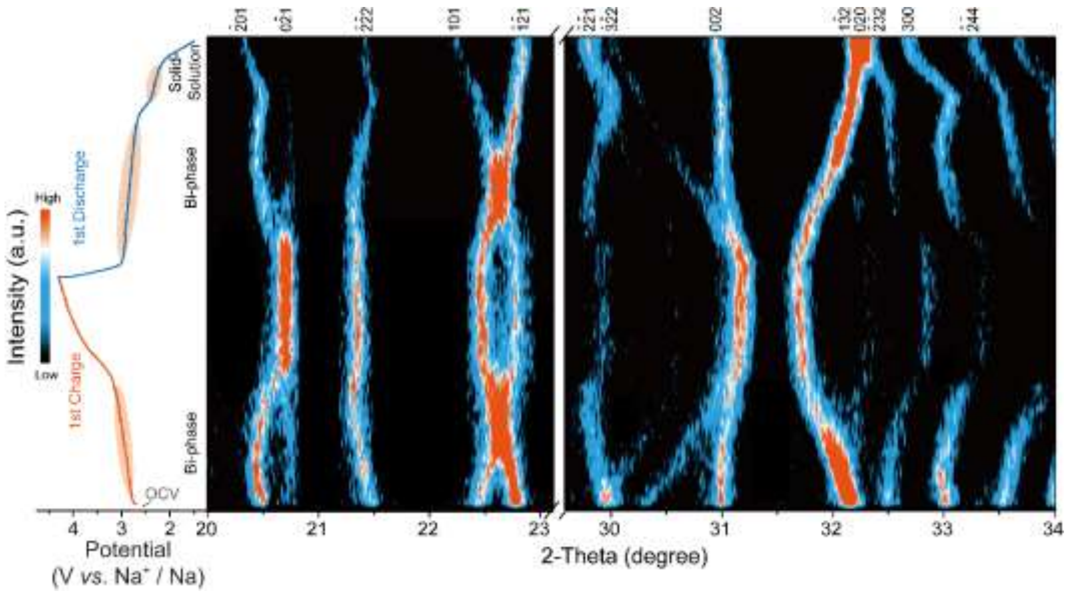

Figure S22. In situ XRD pattern of NFPO-D during the first cycle.

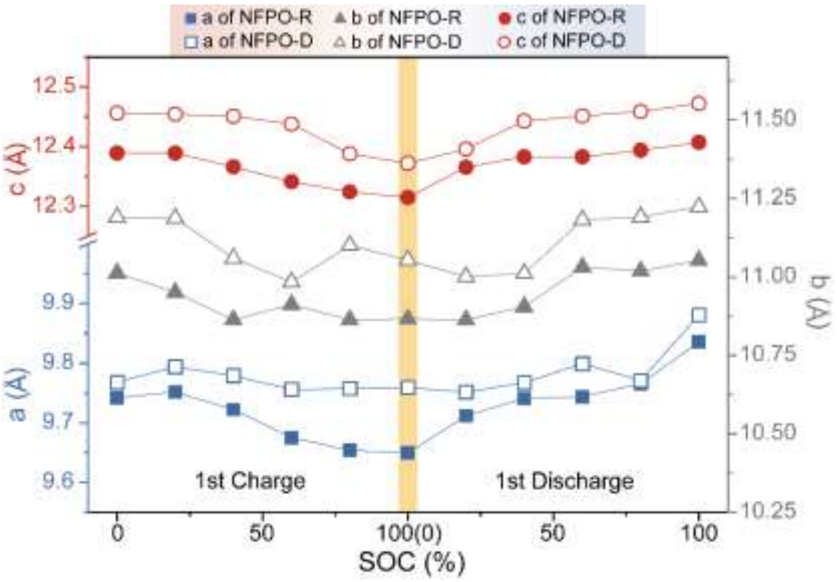

Figure S23. Cell parameters variation during the first cycle.

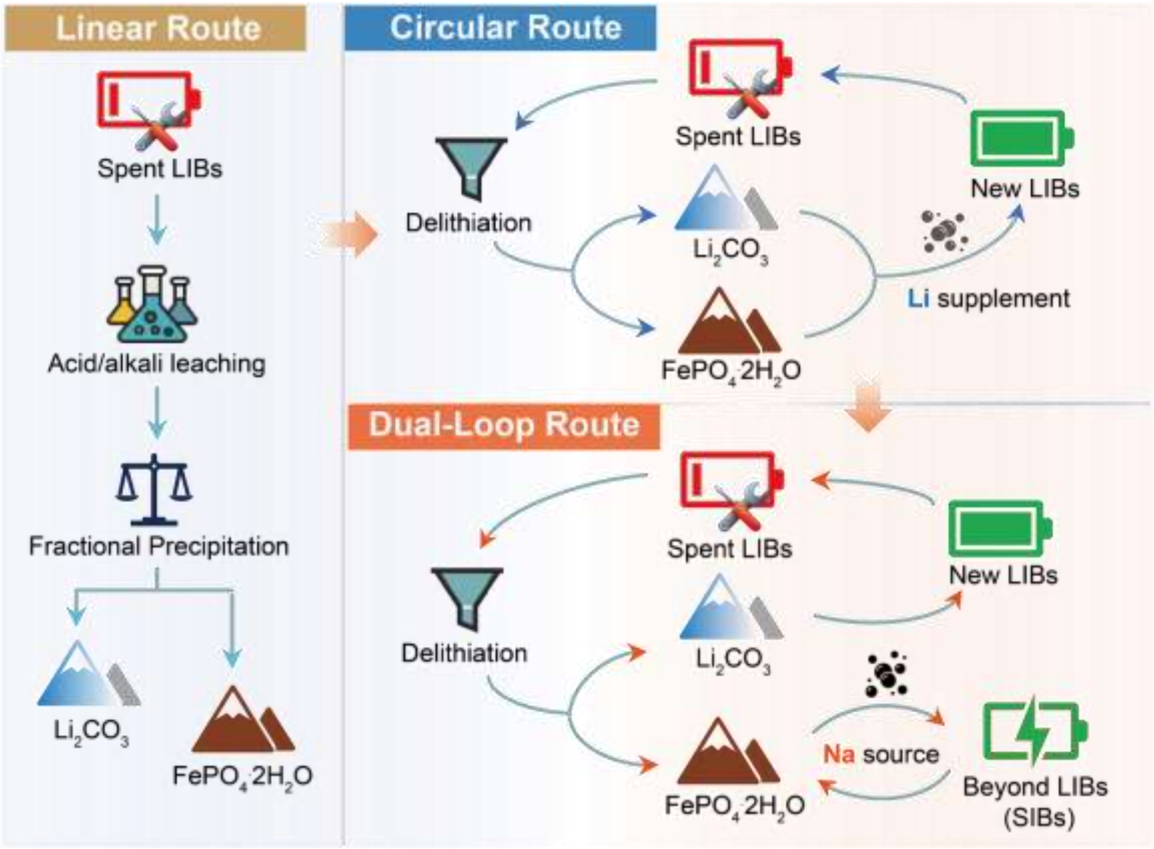

Figure S24. Schematic flow diagrams of recycling spent LFP batteries along linear routes, circular recycling route and dual-loop route.

Table S1. Refined XRD structure information of NFPO-R.

Crystal phase: trigonal, *P-1* (S. G.)						
Atom	x	y	z	Occ.	U	Wyckoff Position
Fe1	0.72105	0.39848	0.21740	0.953	0.025	2i
Fe2	0.64467	-0.00059	0.26450	0.994	0.022	2i
P1	0.57530	0.21143	0.45238	1.000	0.021	2i
P2	0.12784	0.04303	0.31836	1.000	0.034	2i
P3	0.06455	0.33053	0.79029	1.000	0.019	2i
P4	0.28734	0.69213	0.03579	1.000	0.012	2i
Na1	0.02498	0.31437	0.47997	1.000	0.004	2i
Na2	0.41229	0.65368	0.32550	0.982	0.034	2i
Na3	0.21741	0.34225	0.09486	0.955	0.042	2i
Na4	0.50000	0.00000	0.00000	0.395	0.009	1d
Na5	0.26420	0.00566	0.02563	0.409	0.283	2i
Na6	0.49330	-0.01460	0.04942	0.282	0.051	2i
O1	0.01466	0.22030	0.29777	1.000	0.028	2i
O2	0.65571	0.35787	0.42275	0.999	0.045	2i
O3	0.30372	0.02921	0.22599	0.928	0.030	2i
O4	0.11527	0.43447	0.84038	0.993	0.037	2i
O5	0.75484	0.04343	0.53958	0.929	0.018	2i
O6	0.27878	0.18378	0.80927	1.000	0.017	2i
O7	0.02690	0.04152	0.69113	0.944	0.003	2i
O8	0.78077	0.40894	0.03011	0.978	0.005	2i
O9	0.43674	0.57047	0.16836	0.978	0.006	2i
O10	0.62332	0.17257	0.05313	1.000	0.024	2i
O11	0.37123	0.19198	0.53946	0.947	0.017	2i
O12	0.56018	0.19610	0.31873	0.963	0.021	2i
O13	0.08523	0.76557	0.10136	0.991	0.009	2i
O14	0.03341	0.59100	0.34898	0.982	0.011	2i

Table S2. Refined XRD structure information of NFPO-D.

Crystal phase: triclinic, *P-1* (S. G.)						
Atom	x	y	z	Occ.	U	Wyckoff Position
Fe1	0.72600	0.39045	0.21893	1.000	0.025	2i
Fe2	0.64466	-0.00093	0.26350	0.962	0.022	2i
P1	0.57437	0.21406	0.45602	1.001	0.021	2i
P2	0.12761	0.03882	0.31855	1.001	0.034	2i
P3	0.06418	0.33270	0.78889	1.001	0.019	2i
P4	0.29179	0.69296	0.03811	1.001	0.012	2i
Na1	0.02539	0.31715	0.47598	1.001	0.004	2i
Na2	0.39283	0.65264	0.32451	1.001	0.034	2i
Na3	0.20477	0.34712	0.09577	0.997	0.042	2i
Na4	0.50000	0.00000	0.00000	0.283	0.009	1d
Na5	0.25181	0.02804	0.01290	0.498	0.283	2i
Na6	0.47291	-0.00930	0.04177	0.321	0.051	2i
O1	-0.00120	0.21867	0.30443	0.952	0.028	2i
O2	0.65437	0.34800	0.43227	1.001	0.045	2i
O3	0.31173	0.02153	0.22972	1.001	0.030	2i
O4	0.13276	0.39692	0.86520	0.906	0.037	2i
O5	0.75391	0.02073	0.55016	0.949	0.018	2i
O6	0.29016	0.20142	0.79366	0.952	0.017	2i
O7	0.01562	0.03869	0.69238	1.001	0.003	2i
O8	0.78780	0.38553	0.03571	1.001	0.005	2i
O9	0.43778	0.58209	0.15799	0.903	0.006	2i
O10	0.61833	0.17442	0.05413	1.001	0.024	2i
O11	0.37951	0.18921	0.53434	0.799	0.017	2i
O12	0.55671	0.19413	0.31450	0.777	0.021	2i
O13	0.08101	0.76322	0.11012	0.955	0.009	2i
O14	0.02060	0.59805	0.34340	0.925	0.011	2i

Table S3. Element content analysis of NFPO-R and NFPO-D by ICP-OES.

	Na (wt%)	Fe (wt%)	P (wt%)
NFPO-R	10.8777	17.0623	17.0790
NFPO-D	13.5770	21.6890	17.9970

Table S4. EXAFS fitting parameters at the Fe *K*-edge various samples (S_0^2=0.80).

samples	path	C. N. [a]	R (Å) [b]	σ^2 ($\times 10^{-3}$ Å^2) [c]	ΔE (eV) [d]	R factor [e]
NFPO-R	Fe1-O	4.2±0.6	2.00±0.02	7.8±1.9	-0.9±2.9	0.01
	Fe2-O	1.0±0.5	2.14±0.02	2.7±2.0		
	Fe1-O-P	1.1±0.2	2.73±0.03	5.2±1.8		
	Fe2-O-P	1.5±0.8	3.26±0.03	4.7±3.8		
NFPO-D	Fe1-O	4.4±0.5	2.00±0.02	5.2±3.8	-2.4±1.4	0.01
	Fe2-O	0.9±0.4	2.14±0.02	6.9±5.4		
	Fe1-O-P	1.2±0.2	2.85±0.03	3.0±2.7		
	Fe2-O-P	1.6±0.3	3.30±0.03	8.3±5.4		

[a] C. N.: coordination numbers; [b] *R*: bond distance; [c] σ^2: Debye-Waller factors; [d] ΔE_0: the inner potential correction. [e] *R* factor: goodness of fit.

Table S5. Electronic structural information of NFPO-R.

Spin Channel	Up	Down	Total
Band Gap (eV)	3.2720	0.0030	0.0030
Eigenvalue of VBM (eV)	2.9395	4.1666	4.1666
Eigenvalue of CBM (eV)	6.2115	4.1695	4.1695
Fermi Energy (eV)	4.1676	4.1676	4.1676
Highest-Occupied Band	153	137	137
Lowest-Occupied Band	154	137	137
Location of VBM (up)	0.0000	0.0000	0.5000
Location of CBM (up)	0.0000	0.0000	0.0000
Location of VBM (down)	0.0000	0.0000	0.2778
Location of CBM (down)	0.0000	0.2222	0.0000
Location of VBM (total)	0.0000	0.0000	0.2778
Location of CBM (total)	0.0000	0.2222	0.0000

Table S6. Electronic structural information of NFPO-D.

Spin Channel	Up	Down	Total
Band Gap (eV)	3.5240	0.2801	0.2801
Eigenvalue of VBM (eV)	2.6836	4.0514	4.0514
Eigenvalue of CBM (eV)	6.2076	4.3314	4.3314
Fermi Energy (eV)	4.0924	4.0924	4.0924
Highest-Occupied Band	153	137	137
Lowest-Occupied Band	154	138	138
Location of VBM (up)	0.0000	0.0000	0.5000
Location of CBM (up)	0.0000	0.0000	0.0000
Location of VBM (down)	0.0000	0.0000	0.5000
Location of CBM (down)	0.0000	0.5000	0.0000
Location of VBM (total)	0.0000	0.0000	0.5000
Location of CBM (total)	0.0000	0.5000	0.0000

Table S7. Price of the involved materials.

Material	M (g/mol)	Price ($/kg) [a]	Data Sources
Black mass	157.76	1.83	https://www.smm.cn/
H_2SO_4	98.076	0.16	https://www.100ppi.com/
H_2O_2 (30 wt%)	34.016	1.23	https://www.100ppi.com/
Na_2CO_3	106.08	0.36	https://www.100ppi.com/
NaH_2PO_4	119.976	0.76	https://www.100ppi.com/
NaOH	39.998	0.50	https://guba.eastmoney.com/
Li_2CO_3	73.89	12.02	https://www.smm.cn/
$LiFePO_4$	157.76	6.89	https://www.smm.cn/
$Na_2FeP_2O_7$	275.77	3.99	https://detail.1688.com/
$Na_2S_2O_8$	238.104	0.76	https://www.100ppi.com/
Citric acid	192.12	0.66	https://www.100ppi.com/
Glucose	342.296	0.55	https://www.100ppi.com/
$Na_4P_2O_7$	265.9	0.69	https://detail.1688.com/

[a] Based on the exchange rate of 1 yuan = 0.1372 dollars.

Table S8. Life cycle inventories (LCIs) for recycling 1kg LFP black mass *via* linear route.

Section	Material	Amount	Unit	Corresponding LCI	Distribution
Input	Black mass	1	kg	Cathode, LFP, for Li-ion battery {CN}| cathode production, LFP, for Li-ion battery | Cut-off, S	Lognormal
	H_2SO_4	2.013	kg	Sulfuric acid {RoW}| market for sulfuric acid | Cut-off, S	Lognormal
	H_2O_2	2.119	kg	Hydrogen peroxide, without water, in 50% solution state {RoW}| market for hydrogen peroxide, without water, in 50% solution state | Cut-off, S	Lognormal
	Na_2CO_3	1.244	kg	Sodium bicarbonate {GLO}| market for sodium bicarbonate | Cut-off, S	Lognormal
	NaH_2PO_4	0.630	kg	Sodium phosphate {RoW}| market for sodium phosphate | Cut-off, S	Lognormal
	NaOH	0.561	kg	Sodium hydroxide, without water, in 50% solution state {GLO}| market for sodium hydroxide, without water, in 50% solution state | Cut-off, S	Lognormal
	Water	20.75	kg	Water, decarbonized {CN}| market for water, decarbonized | Cut-off, S	Lognormal
	Heat	2.67	MJ	Heat, from stream, in chemical industry {RoW}| market for heat, from stream, in chemical industry | Cut-off, S	Lognormal
	Electricity	10.63	kWh	Electricity, low voltage {CN-CCG}| electricity voltage transformation from medium to low voltage | Cut-off, S	Lognormal
Output	Wastewater	20.75	kg	Wastewater, average {RoW}| market for wastewater, average | Cut-off, S	Lognormal
	Solid waste	0.182	kg	Municipal solid waste	Lognormal

				{RoW} | market for municipal solid waste | Cut-off, S	
	CO_2	0.26	kg	Emissions to air	Lognormal
Product	Li_2CO_3	0.19	kg	/	/

Table S9. LCIs for recycling 1kg LFP black mass *via* circular route.

Section	Material	Amount	Unit	Corresponding LCI	Distribution
Input	Black mass	1	kg	Cathode, LFP, for Li-ion battery {CN}| cathode production, LFP, for Li-ion battery | Cut-off, S	Lognormal
	$Na_2S_2O_8$	2.055	kg	Sodium persulfate {GLO} | market for sodium persulfate | Cut-off, S	Lognormal
	Glucose	0.218	kg	Glucose {GLO} | market for glucose | Cut-off, S	Lognormal
	Na_2CO_3	0.151	kg	Sodium bicarbonate {GLO}| market for sodium bicarbonate | Cut-off, S	Lognormal
	Li_2CO_3	0.051	kg	Lithium carbonate {GLO}| market for lithium carbonate | Cut-off, S	Lognormal
	Water	15.23	kg	Water, decarbonized {CN}| market for water, decarbonized | Cut-off, S	Lognormal
	Electricity	3.88	kWh	Electricity, low voltage {CN-CCG}| electricity voltage transformation from medium to low voltage | Cut-off, S	Lognormal
	Heat	3.26	MJ	Heat, from stream, in chemical industry {RoW}| market for heat, from stream, in chemical industry | Cut-off, S	Lognormal
Output	Wastewater	15.23	kg	Wastewater, average {RoW}| market for wastewater, average | Cut-off, S	Lognormal
	Solid waste	0.223	kg	Municipal solid waste {RoW} | market for municipal solid waste | Cut-off, S	Lognormal
	CO_2	0.33	kg	Emissions to air	Lognormal
Product	$LiFePO_4$	0.9	kg	/	/

Table S10. LCIs for recycling 1kg LFP black mass *via* dual-loop route.

Section	Material	Amount	Unit	Corresponding LCI	Distribution
Input	Black mass	1	kg	Cathode, LFP, for Li-ion battery {CN}| cathode production, LFP, for Li-ion battery | Cut-off, S	Lognormal
	$Na_2S_2O_8$	2.055	kg	Sodium persulfate {GLO} | market for sodium persulfate | Cut-off, S	Lognormal
	$Na_4P_2O_7$	0.840	kg	Sodium pyrophosphate {GLO}| market for sodium pyrophosphate | Cut-off, S	Lognormal
	Citric acid	0.607	kg	Citric acid {GLO} | market for citric acid | Cut-off, S	Lognormal
	Na_2CO_3	0.151	kg	Sodium bicarbonate {GLO}| market for sodium bicarbonate | Cut-off, S	Lognormal
	Water	15.23	kg	Water, decarbonized {CN}| market for water, decarbonized | Cut-off, S	Lognormal
	Electricity	3.41	kWh	Electricity, low voltage {CN-CCG}| electricity voltage transformation from medium to low voltage | Cut-off, S	Lognormal
	Heat	2.73	MJ	Heat, from stream, in chemical industry {RoW}| market for heat, from stream, in chemical industry | Cut-off, S	Lognormal
Output	Wastewater	15.23	kg	Wastewater, average {RoW}| market for wastewater, average | Cut-off, S	Lognormal
	Solid waste	0.101	kg	Municipal solid waste {RoW} | market for municipal solid waste | Cut-off, S	Lognormal
	CO_2	0.28	kg	Emissions to air	Lognormal
Product	$Na_2FeP_2O_7$	1.74	kg	/	/
	Li_2CO_3	0.21	kg	/	/

Table S11. Comparison of environmental characterization among three methods in various impact categories.

Impact Category	Unit	Linear Route	Circular Route	Dual-Loop
Global warming, Human Health	kg CO_2-Eq	0.000138	0.000103	9.53E-5
Global warming, Terrestrial ecosystems	kg CO_2-Eq	4.17E-7	3.12E-7	2.88E-7
Global warming, Freshwater ecosystems	kg CO_2-Eq	1.14E-11	8.52E-12	7.85E-12
Stratospheric ozone depletion	kg CFC-11-Eq	1.92E-7	1.43E-7	1.41E-7
Ionizing radiation	kBq Co-60-Eq	4.36E-8	3.48E-8	3.16E-8
Ozone formation, Human health	kg NO_x-Eq	3.28E-7	2.47E-7	2.25E-7
Fine particulate matter formation	kg PM2.5-Eq	0.000159	0.000128	0.000108
Ozone formation, Terrestrial ecosystems	kg NO_x-Eq	4.78E-8	3.6E-8	3.29E-8
Terrestrial acidification	kg SO_2-Eq	1.39E-7	1.24E-7	8.97E-8
Freshwater eutrophication	kg P-Eq	1.84E-6	1.35E-6	1.35E-6
Marine eutrophication	kg N-Eq	1.66E-9	1.22E-9	1.22E-9
Terrestrial ecotoxicity	kg 1,4-DCB-Eq	7.93E-9	6.35E-9	5.43E-9
Freshwater ecotoxicity	kg 1,4-DCB-Eq	1.29E-8	9.75E-8	9.2E-9
Marine ecotoxicity	kg 1,4-DCB-Eq	2.65E-9	2E-9	1.9E-9
Human carcinogenic toxicity	kg 1,4-DCB-Eq	7.81E-5	5.7E-5	5.53E-5
Human non-carcinogenic toxicity	kg 1,4-DCB-Eq	0.000225	0.000167	0.000163
Land use	m^2*a crop-Eq	2.97E-8	2.46E-8	2.1E-8
Mineral resource scarcity	kg Cu-Eq	0.292	0.243	0.2
Fossil resource scarcity	kg oil-Eq	8.15	6.32	5.71
Water consumption,	m^3	-0.00181	-0.00133	-0.00133

Human health				
Water consumption, Terrestrial ecosystem	m^3	-1.1E-5	-8.09E-6	-8.1E-6
Water consumption, Aquatic ecosystems	m^3	-4.92E-10	-3.61E-10	-3.62E-10

SI References

1. Kresse G, Furthmuller J. Efficiency of *ab-Initio* total energy calculations for metals and semiconductors using a plane-wave basis set. *Comput Mater Sci* 1996; **6**: 15-50.
2. Kresse G, Furthmuller J. Efficient iterative schemes for *ab Initio* total-energy calculations using a plane-wave basis set. *Phys Rev B* 1996; **54**: 11169-86.
3. Perdew JP, Burke K, Emzerhof M. Generalized gradient approximation made simple. *Phys Rev Lett* 1996; **77**: 3865-8.
4. Perdew JP, Ernzerhof M, Burke K. Rationale for mixing exact exchange with density functional approximations. *J Chem Phys* 1996; **105**: 9982-5.
5. Kresse G, Joubert D. From ultrasoft pseudopotentials to the projector augmented-wave method. *Phys Rev B* 1999; **59**: 1758-75.
6. Gu Z-Y, Zhao X-X, Li K, et al. Homeostatic solid solution reaction in phosphate cathode: breaking high-voltage barrier to achieve high energy density and long life of sodium-ion batteries. *Adv Mater* 2024; **36**: 2400690.
7. Gu Z-Y, Guo J-Z, Zhao X-X, et al. High‐ionicity fluorophosphate lattice *via* aliovalent substitution as advanced cathode materials in sodium‐ion batteries. *InfoMat* 2021; **3**: 694-704.
